# Surgical Techniques for Radical Trachelectomy

**DOI:** 10.3390/cancers17060985

**Published:** 2025-03-14

**Authors:** Sebastian Szubert, Magdalena Nadolna, Paweł Wawrzynowicz, Agnieszka Horała, Julia Kołodziejczyk, Łukasz Koberling, Paweł Caputa, Mikołaj Piotr Zaborowski, Ewa Nowak-Markwitz

**Affiliations:** 1 Division of Gynaecological Oncology, Department of Gynaecology, Poznan University of Medical Sciences, 61-701 Poznań, Poland; julia.faustyna.kolodziejczyk@gmail.com (J.K.); lukasz.koberling@gmail.com (Ł.K.); mzaborowski@ump.edu.pl (M.P.Z.); 2 Institute of Bioorganic Chemistry, Polish Academy of Sciences, Noskowskiego 12/14, 61-704 Poznań, Poland

**Keywords:** radical trachelectomy, cervical cancer, uterine artery preservation, nerve-sparing technique, abdominal cerclage, fertility-sparing treatment

## Abstract

**Background/Objectives**: The primary aim of this systematic review was to evaluate fertility outcomes and the oncological safety of different surgical techniques of radical trachelectomy (RT). **Methods**: The systematic review was performed in accordance with the Preferred Reporting Items for Systematic Reviews and Meta-Analyses (PRISMA) guidelines. A systematic literature search on PubMed, Embase, and Google Scholar was performed between 1 November 2023 and 31 March 2024 with no limits for the time of publication. **Results**: In total, 56 studies met the inclusion criteria: 22 for abdominal RT (1712 patients), 14 for endoscopic RT (445 patients), and 22 for vaginal RT (1158 patients). Data regarding certain steps of the procedure (uterine artery preservation, autonomous nerve-sparing, abdominal cerclage, types of sutures used for the cerclage, uterine dilatation during cerclage placement, prolongation of uterine catheterization, type of uterovaginal anastomosis, antibiotic prophylaxis, and suppression of menstruation) were extracted and analyzed with regard to the obstetrical and oncological outcomes. Endoscopic RT was associated with a significantly higher pregnancy rate and a lower rate of preterm deliveries. Uterine artery preservation was associated with a higher live birth rate. Nerve-sparing RT resulted in a higher pregnancy rate, but no differences in the attempt for pregnancy and live birth rates were observed. **Conclusions**: Taking into account the obstetrical outcomes, it seems that the preferred option for radical RT is an endoscopic procedure with preservation of the uterine artery and the pelvic autonomic nerves. However, the safety of the endoscopic approach should be evaluated in prospective trials.

## Simple Summary

Radical trachelectomy (RT) is a fertility-sparing treatment for a selected population with early-stage cervical cancer (CC). In this systematic review, different surgical approaches (open/endoscopic/vaginal) and certain surgical steps of RT were analyzed in terms of fertility and oncological outcomes. Among the 118 studies selected for full reading, 56 studies, including 3315 patients, met the inclusion criteria. All surgical approaches had similar live birth rates and cancer recurrence rates. However, the endoscopic approach was associated with a higher pregnancy rate and a lower preterm delivery rate. Uterine artery preservation resulted in a higher live birth rate, and the nerve-sparing technique resulted in a higher pregnancy rate. Although obstetrical outcomes were the best after endoscopic RT, the safety of this procedure should be assessed in prospective trials. Uterine artery-sparing and nerve-sparing techniques seem to have better obstetrical outcomes and should be considered during RT.

## 1. Introduction

The incidence of cervical cancer (CC) is estimated to be over 600,000 cases yearly, resulting in about 342,000 deaths. World’s CC incidence rate is 13.3/100,000 [1]. Although screening tests help to detect and cure premalignant disease and thus reduce the incidence of CC, it remains the fourth most common cancer in women [1]. CC is most frequently diagnosed worldwide among women aged 50–55 [2], but in developed countries like the USA, nearly 15% and 25% of patients are diagnosed in the age ranges 20–34 and 35–44, respectively [3]. Taking into account the rising mean age of having the first child in developed countries, an increasing number of women might be interested in fertility-sparing CC treatment options.

Radical trachelectomy (RT) is a fertility-sparing treatment method in early-stage (IB1-IB2) cervical cancer. During the surgery, the cervix with the tumor, the upper part of the vagina, the parametria, and the pelvic lymph nodes are removed. This extent of the surgery is needed to avoid the potential residue of cancer cells, which can cause recurrences [4]. RT was introduced as an alternative to radical hysterectomy (RH) in patients who wished to preserve their fertility, as in RT, the uterine corpus is preserved. Recently, RT has been replaced by cervical conization more frequently [5]. However, according to the National Comprehensive Cancer Network (NCCN), conization for treating CC can only be considered if the following criteria are met: negative surgical margins after conization, tumor size less than 2 cm, depth of invasion ≤ 10 mm, squamous cell carcinoma or usual type adenocarcinoma grade 1 or 2 and no lympho-vascular space invasion (LVSI) [6]. Invasion of lymphatic vessels by tumor cells can result in cancer spreading beyond the cervix. The dominant route of CC dissemination leads through the parametrial lymphatic channels to the pelvic lymph nodes. Therefore, the presence of LVSI requires the removal of the parametria and the corresponding lymph nodes to ensure radical treatment of CC. Although RT is mainly performed in tumors less than 2 cm, several reports suggest that the surgery may also be safe for selected patients with larger tumors and even tumors with vaginal infiltration. However, in these cases, neoadjuvant chemotherapy is often administered [7,8].

Thanks to the organized CC screening and human papillomavirus (HPV) vaccines, CC incidence decreased in developed countries. Therefore, RT is a procedure that is rarely performed. The surgical approach in RT is not standardized. Vaginal, abdominal, laparoscopic, and robotic approaches have been employed during this procedure. Moreover, the extent of surgery may be different. Contentious issues involve preservation of the uterine artery, nerve-sparing technique, cerclage placement, and type of utero-vaginal anastomosis. There is a lack of randomized controlled trials comparing different types of RT. Similarly, data evaluating various surgical techniques for RT are limited. Thus, the primary aim of this systematic review is to evaluate different surgical techniques used in RT in terms of their oncological safety and impact on future fertility. The secondary purpose is to evaluate differences and trends in surgical techniques among different clinicians.

## 2. Materials and Methods

This systematic review complies with the Preferred Reporting Items for Systematic Reviews and Meta-Analyses (PRISMA) guidelines.

### 2.1. Literature Search

We performed a systematic literature search on PubMed, Embase, and Google Scholar (including the first 200 records) using the keywords “radical trachelectomy”. The search was restricted to articles written in English. All searches were conducted between 1 November 2023 and 31 March 2024. Three authors (MN, PW, and JK) independently screened the titles of the studies and prepared a list of potentially relevant articles. Then, duplicates were removed, and four authors (SS, MN, PW, and JK) analyzed the titles and abstracts of the articles together and chose the articles for further analysis. Next, the senior authors (SS and AH) analyzed the chosen articles to select those for full reading and data extraction. Only the following types of studies were included: experimental clinical studies, observational studies, case series, cohort studies, case–control studies, and cross-sectional studies. The following studies were excluded: single case reports, registry analyses, reviews, meta-analyses, and conference abstracts. Studies that used a single surgical approach to RT were included in the final analysis. Articles with mixed surgical approaches were only included if data regarding particular techniques could be separated. Studies concerning the role of conization in fertility-sparing treatment of cervical cancer were also excluded. Studies on trachelectomy performed during pregnancy were excluded unless they also provided data for non-pregnant patients that could be separately analyzed. The same rule was applied to neoadjuvant chemotherapy; the study was included in the final analysis only if the data concerning cases without neoadjuvant chemotherapy could be extracted.

### 2.2. Data Extraction and Synthesis

Four authors (SS, MN, PW, and JK) read and extracted the following data from the selected articles: type of surgical approach (1) laparotomy, (2) endoscopic (minimally invasive surgery, MIS, including laparoscopy and robotic), and (3) vaginal approach), histopathological type of the tumor, median time from surgery to pregnancy, surgery adverse events, attempt for pregnancy rate, pregnancy rate, live birth rate, preterm delivery rate, and recurrence rate. The ‘pregnancy rate’ was defined as the rate of patients who became pregnant. The ‘attempt for pregnancy rate’ was defined as the rate of patients who declared the intention to conceive. The ‘live birth rate’ was defined as the rate of live births after 24 weeks of gestation among all patients who underwent RT. Then, we calculated the ‘pregnancy rate among attempts’, which was defined as the pregnancy rate among patients who actually attempted to become pregnant. The ‘preterm delivery rate’ was calculated as the rate of deliveries after 24 and before 37 weeks of gestation among all live births. Some authors reported ongoing pregnancies in their studies. Given the lack of data on the outcome of uncompleted pregnancies, these patients were excluded from the calculation of the live birth rate and preterm delivery rate.

The stage of the disease was reported according to the International Federation of Gynecology and Obstetrics (FIGO) classification from 2018. In the case of the studies published using earlier versions of the FIGO classification, the IB tumors were restaged according to the 2018 version based on the tumor size. However, when the tumor size was not available, the classification was reported as in the original study; however, the data were excluded from the statistical comparisons between the FIGO stages. Due to no significant changes in the classification of IA tumors, the tumors were not reclassified and reported as in original studies.

The studies were divided into three groups based on the surgical approach: (i) group 1—abdominal RT; (ii) group 2—endoscopic (MIS, both laparoscopic and robotic) RT; (iii) group 3—vaginal RT. The number of articles using different surgical procedures was assessed to evaluate the general trends among surgeons. Next, the data concerning the analyzed procedures were summarized to evaluate oncological and obstetrical outcomes.

Data on the following steps of RT were extracted and evaluated: uterine artery preservation, nerve-sparing surgery, placement of abdominal cerclage, type of suture used for the cerclage, uterine dilatation during cerclage placement, prolongation of uterine catheterization, type of uterovaginal anastomosis, antibiotic prophylaxis, and suppression of menstruation. Data concerning nerve-sparing surgery were evaluated only in the case of abdominal and minimally invasive RT because this technique is not feasible during vaginal RT. Only the data from articles that clearly stated the technique used (uterine artery- and nerve-sparing or non-sparing) were analyzed. Otherwise, the data were regarded as not available.

Regarding surgery-related morbidity, the following adverse events were evaluated: cervical stenosis, infectious complications (wound infection, febrile morbidity, vaginal discharge, pelvic peritonitis, and formation of pelvic abscess), lymphatic complications (lymphoceles, lower limb edema, pelvic lymph seroma, temporary vulvar edema), secondary infertility (fallopian tube obstruction, Asherman syndrome, and amenorrhea); urological adverse events (urinary bladder dysfunction, stress incontinence, urinary retention, lower urinary tract infection, bladder injury, and fistula formation), and vaginal bleeding.

Only high-quality articles, in which the authors provided a clear description of surgical management and its results, were analyzed. When the description of the technique was insufficient to extract the data according to the criteria described above, the whole study or the data concerning analyzed element of the surgical technique for trachelectomy was excluded from the analysis. Some authors reported their outcomes in a few subsequent articles. In such situations, the data regarding oncological and obstetrical outcomes were extracted from the last published article. However, the data regarding surgical techniques were also extracted from the previous reports.

### 2.3. Statistical Analysis

The data extracted from the analyzed studies were used for the preparation of contingency tables. Nominal data comparisons between the groups were made using the Fisher-exact test. The Freeman–Halton extension was used for 3 × 2 contingency tables. In the case of a large sample size (above 300) in a 3 × 2 contingency table, the Chi-square test for trend was used. The relationship between the date of study publication and the techniques used for RT was investigated using Spearman correlation.

Statistical analysis was conducted using MedCalc 11.4.2.0., MedCalc Software, Seoul, Republic of Korea, and GraphPad In Stat 3.06, GraphPad Software Inc., San Diego, CA, USA.

## 3. Results

### 3.1. Overview of the Studies

**Figure 1:**
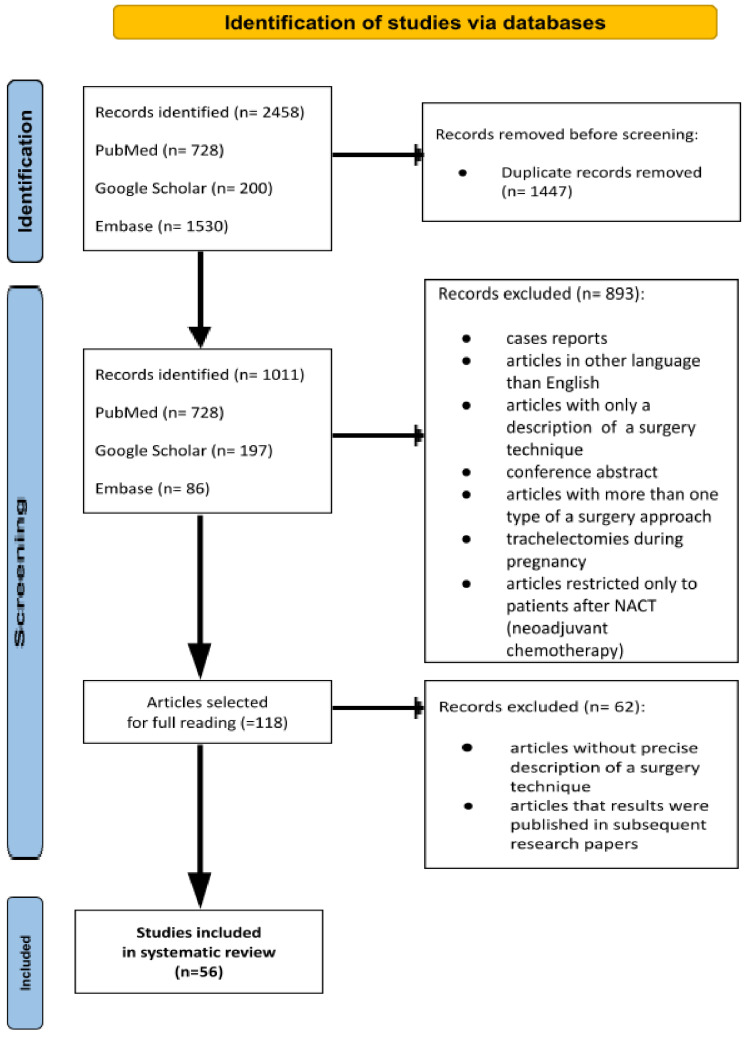
Flowchart of the article selection process.

The article selection process is presented in Figure 1. Data from all the studies are summarized in Table 1, Table 2 and Table 3.

One hundred eighteen studies were selected for full reading. Then, 56 articles that met the inclusion criteria were identified. One article [9] on the abdominal technique was divided into two independent ones (because of using modified type II and type III RT techniques among patients). The data from an article comparing both abdominal and vaginal approaches [10] were extracted and separated to evaluate abdominal and vaginal approaches independently. Finally, data from 22, 14, and 22 studies concerning abdominal, endoscopic, and vaginal radical trachelectomy, respectively, were included for statistical analysis.

Endoscopic surgeries included both laparoscopic and robotic surgery. The article selection process is demonstrated in the flowchart (Figure 1). The list of selected articles with analyzed data is presented in Table 1, Table 2 and Table 3.

**Table 1:** The list of the articles and extracted data concerning abdominal technique for radical trachelectomy.

Abdominal																				
Study	Number of Cases	FIGO	Tumor Types	Technique									Median Time to Pregnancy	IVF Rate (%)	Attempt for Pregnancy (%)	Pregnancy Rate/Attempt for Pregnancy (%)	Pregnancy Rate/Number of Patients Wit RT (%)	Live Birth Rate (%)	Preterm Delivery Rate (%)	Recurrence Rate (%)
				Uterine Artery Preservation	Nerve Sparing Surgery	Abdominal Cerclage	Type of Suture (Abdominal Cerclage)	Uterine Dilatation During Cerclage Placement	Prolongation of Uterine Catheterization	Type of Utero-Vaginal Anastomosis	Extended Antibiotic Prophylaxis	Suppression of Menstruation								
Kiss S.L. et al. [11]; 2021	18	FIGO *: stage IA2 (6) to IB1 (4), IB2 (5), IB3 (4); >2 cm	SCC (15), AC (2) or ASC (1),	no	N/A	no	no	N/A	no	interrupted or a continuous absorbable suture	no	N/A	N/A	N/A	7/18 (38.9%)	3/7 (42.9%)	3/18 (16.67%)	5/5 (100%)	0 (0%)	2/18 (11.11%)
Wang Y. et al. [12]; 2020	31	FIGO *: IA1 (1), IA2 (6), IB1 (24)	SCC (29) and AC (2)	yes	N/A	yes	polypropylene mesh	yes	Foley catheter (8 weeks)	N/A	N/A	no	N/A	2/5	15/31 (48.4%)	5/15 (33.3%)	5/23 (21.7%)	3/5 (60%)	2/3 (66.67%)	2/31 (9.7%)
Mabuchi S. et al. [13]; 2017	3	FIGO *: IA2 (1), IB1 (2)	SCC (3),	yes	yes	yes	polyester suture size #0	N/A	Foley catheter	N/A	no	N/A	N/A	N/A	N/A	N/A	N/A	N/A	N/A	0/3 0%
Li X. et al. [14]; 2016	107	FIGO *: IB1 (46); IB2 (61)	SCC (91), AC (14), other (2);	no	no	yes	polyester Mersilene tape	N/A	T-IUD	interrupted, absorbable sutures	N/A	N/A	N/A	2/7 (28.57%)	38/107 (35.51%)	7/38 (18.4%)	7/107 (6.5%)	6/7 (85.71%)	1/6 (16.7%)	2/107 (1.87%)
van Gent M.D. et al. [15]; 2014	28	FIGO **: IA1 (3), IB1 (22), IB2 (3); ~1.73 cm	SCC (14), AC (11), other (3);	yes	yes	yes	N/A	yes	Foley catheter (7 days)	interrupted, absorbable sutures	yes	N/A	N/A	2/26 (7.7,%)	17/26 (65,4%)	9/17 (52.9%)	9/28 (3.21%)	9/9 (100%)	0/14 (0%)	2/28 (7.14%)
Lintner B. et al. [16]; 2013	31	FIGO **: IB2 (17), IB3 (14); 2–4 cm	SCC (17), AC (4), other (10);	no	no	yes	N/A	N/A	N/A	interrupted, absorbable sutures	N/A	N/A	N/A	N/A	8/31 (25.8%)	4/8 (50%)	4/31 (12.9%)	3/4 (75%)	1/3 (33.33%)	4/31 (12.9%)
Muraji M. et al. [9]; type III; 2012	8	FIGO *: IB1 (8)	SCC (5) AC (3),	yes	no	N/A	N/A	N/A	N/A	1-0 polydioxanone suture	N/A	N/A	N/A	0 (0%)	N/A	1 (not defined which group)	N/A	1/1 (100%)	1/1 (100%)	0/8 (0%)
Muraji M. et al. [9], type II with nerve- sparing; 2012	15	FIGO *: IB1 (2) IA2 (2) IB1 (11)	SCC (11), AC (3), other (1)	yes	yes	N/A	N/A	N/A	N/A	N/A	N/A	N/A	N/A	N/A	N/A	1 (not defined which group)	N/A	N/A	N/A	0/15 (0%)
Wethington S.L. et al. [17]; 2012	81	FIGO **: IA1-3, IA2 (8,) IB1 (88), IB2 (1), IIA (1)	SCC (40), AC (54), ASC	no	N/A	yes	N/A	N/A	N/A	N/A	N/A	N/A	N/A	N/A	38/70 (54.3%)	28/38 (73.7%)	28/81 (34.57%)	16/25 (64%)	N/A	4/81 (4.94%)
Karateke A. et al. [18]; 2012	8	FIGO *: IB1 (2), IB2 (5), IIA (1)	SCC (4), AC (3), other (1), >2 cm (5) <2 cm (3)	no	N/A	no	N/A	no	N/A	interrupted, absorbable sutures	no	N/A	N/A	N/A	N/A	N/A	3/8 (37.5%)	(2/3) 66.67%	2/3 (66.67%)	0/8 (0%)
Li J. et al. [19]; 2011	59	FIGO *: IB1 (2), IB2 (5), IIA (1)	SCC (40) (4), AC (3), other (1), >2 cm (5) <2 cm (3)	yes	N/A	yes	polyester Mersilene tape	N/A	T-IUD	interrupted, absorbable sutures	N/A	N/A	N/A	N/A	10/56 (17.86%)	2/10 (20%)	N/A	N/A	N/A	0/59 (0%)
Nishio H. et al. [20]; 2009	61	FIGO *: IA1 (4), IA2 (8), IB1 (36), IB2 (13)	SCC (58), AC (2), ASC (1);	yes	N/A	yes	nylon sutures (without size)	N/A	1 (3–4 weeks)	interrupted, absorbable sutures	N/A	N/A	13 months	17/29 (58.62%)	29/61 (47.54%)	4/29 (13.79%)	4/61 (6.56%)	4/4 (100%)	2/4 (50%)	6/61 (9.84%)
Okugawa K. et al. [21]; 2019	182	FIGO *: IA1 (3) IA2 (23) IB1 (94) IB2 (58) IIA1 (4)	SCC (125), AC (46), ASC (11)	yes	yes	yes	N/A	N/A	N/A	N/A	N/A	N/A	N/A	N/A	N/A	N/A	26/182 (14.23%)	20/36 (55.56%)	12/34 (35.29%)	3/182 (1.64%)
Kim C.H. et al. [22]; 2012	77	FIGO *: IA1, IA2, IB1	SCC (45), ASC (10), AC (50);	N/A	N/A	yes	polyester suture size #0	N/A	N/A	N/A	N/A	N/A	6 months	11/23 (47.82%)	35/77 (45.45%)	23/35 (65.71%)	23/77 (29.87%)	20/23 (86.96%)	7/20 (35%)	N/A
Cǎpîlna M.E. et al. [23]; 2014	26	FIGO **: IA2 (11), IB1 (14), IB2 (1)	SCC (15), ASC (8), AC (3)	no	N/A	no	N/A	N/A	N/A	N/A	N/A	no	N/A	0/3 (0%)	7/26 (30.43%)	3/7 (42.85%)	3/26 (11.54%)	1/3 (33.33%)	0 (0%)	1/26 (3.85%)
Li X. et al. [24]; 2019	333	FIGO *: IA1 (50), IA2 (28) IB1 (255)	SCC (271), AC (51), ASC (11)	no	no	yes	polytetrafluoroethylene (ePTFE) GoreTex	N/A	T-IUD	interrupted, absorbable sutures	N/A	N/A	N/A	N/A	N/A	N/A	22/333 (6.61%)	15/22 (68.18%)	3/15 (20%)	11/333 (3.3%)
Nakajima T. et al. [25]; 2020	32	FIGO *: IA1 (3), IB1 (29)	SCC (25), AC (5), other (2);	yes	yes	yes	polyester suture size #0	yes	plastic tube (7 days)	U-shaped sutures	N/A	N/A	N/A	N/A	9/32 (28.12%)	6/9 (66.67%)	6/32 (18.75%)	6/6 (100%)	2/6 (33.33%)	0/32 0%
Li X. et al. [26]; 2020	360	FIGO *: IA1 (50), IA2 (32), IB1 (268)	SCC (285), AC (51), other (24);	N/A	N/A	yes	polytetrafluoroethylene (ePTFE) GoreTex	N/A	N/A	N/A	N/A	N/A	N/A	16/30 (53.33%)	149/360 (41.39%)	26/149 (17.45%)	26/360 (7.22%)	19/26 (73.11%)	5/19 (26.32%)	N/A
Tamauchi S. et al. [27]; 2016	28	FIGO **: IA2 (4), IB1 (24)	SCC (21), AC (5), other (2);	yes	yes	yes	N/A	N/A	N/A	N/A	N/A	no	N/A	5/8 (62.5%)	12/28 (42.85%)	8/12 (66.67%)	8/28 (28.57%)	5/8 (62.5%)	4/5 (80%)	0/28 (0%)
Nishio H. et al. [28]; 2013	114	FIGO **: IA1 (9), IA2 (12), IB1 (93)	SCC (99), AC (14), other (1)	yes	yes	yes	polyester suture size #0	N/A	N/A	N/A	N/A	N/A	32.6 +/− 22 months	20/31 (64.52%)	69/114 (60.53%)	25/69 (36.23%)	25/114 (2.,93%)	21/25 (84%)	17/21 (80.95%)	N/A
Tokunaga H. et al. [29]; 2014	42	FIGO *: IA1 (1), IA2(4), IB1 (37), <2 cm	SCC (42);	N/A	yes	N/A	N/A	N/A	N/A	N/A	N/A	N/A	N/A	9/18 (50%)	18/42 (42.86%)	5/18 (27.78%)	5/42 (11.9%)	3/5 (60%)	2/3 (66.67%)	3/42 (7.14%)
Wang Y. et al. [10]; 2021	68	FIGO *: IA1 (6), IA2 (22), IB (40)	SCC (45), AC (14), other (9);	yes	no	yes	N/A	N/A	Foley catheter	N/A	N/A	N/A	6 months	2/22 (9.1%)	52/68 (76.47%)	11/52 (21.15%)	11/68 (16.18%)	9/11 (81.82%)	3/9 (33.33%)	11/68 (16.18%)

N/A—not available, FIGO *—according to FIGO 2018; FIGO **—according to FIGO 2009; SCC—squamous cell carcinoma; AC—adenocarcinoma; ASC—adenosquamous cell carcinoma; T-IUD—T intrauterine device.

**Table 2:** The list of the articles and extracted data concerning endoscopic technique for radical trachelectomy.

Endoscopic																				
Study	Number of Cases	FIGO	Tumor Types	Technique									Median Time to Pregnancy	IVF Rate (%)	Attempt for Pregnancy (%)	Pregnancy Rate/Attempt for Pregnancy (%)	Pregnancy Rate/Number of Patients Wit RT (%)	Live Birth Rate (%)	Preterm Delivery Rate (%)	Recurrence Rate (%)
				Uterine Artery Preservation	Nerve Sparing Surgery	Abdominal Cerclage	Type of Suture (Abdominal Cerclage)	Uterine Dilatation During Cerclage Placement	Prolongation of Uterine Catheterization	Type of Utero-Vaginal Anastomosis	Extended Antibiotic Prophylaxis	Suppression of Menstruation								
Johansen G. et al. [30]; 2016	48	FIGO **: IA1-IA2 (16), IB1 (32)	SCC (25), ASC (5), AC (18)	no	N/A	yes	N/A	N/A	N/A	N/A	yes	N/A	5.3 year	1/3 (33.3)	10/30 (33.3%)	3/10 (30%)	3/30 (10%)	2/3 (66.6)	0/2 (0%)	3/30 (10%)
Ekdahl L. et al. [31]; 2021	149	FIGO **: IA1 (8), IA2 (29), IB1 (111), IIA (1)	SCC (88), AC (61)	yes	no	yes	polypropylene sutures size #0, polytetrafluoroethylene (ePTFE) GoreTex	N/A	N/A	N/A	N/A	N/A	N/A	17/88 (19.3)	88/149 (59%)	70/88 (79.5%)	70/149 (47%)	76/88 (86.4)	18/76 (23.7%)	11/149 (7.4%)
Xu M. et al. [32]; 2022	12	FIGO *: IA2 (1) IB1 (9) IB2 (2); <2 cm (10) >2 cm (2)	SCC (10), ASC (2)	yes	yes	N/A	N/A	yes	6 months	N/A	N/A	no	N/A	N/A	3/12 (25%)	2/3 (67%)	2/12 (16.67%)	N/A	N/A	0/12 (0%)
Xu M. et al. [33]; 2022	25	FIGO *: IA2 (5), IB1 (16), IB2 (4)	SSC (21), ASC (4)	yes	yes	no	N/A	yes	modified IUD (6 months)	N/A	no	no	N/A	N/A	8/25 (32.0%)	4/8 (50%)	4/25 (16%)	N/A	N/A	N/A
Kanao H. et al. [34]; 2021	35	FIGO *: 1A2 (8), IB1 (29) 2A1 (3)	SCC (29), other (11)	yes	yes	yes	polypropylene sutures size #2	N/A	N/A	N/A	N/A	N/A	N/A	2/9 (22.2)	9/35 (26%)	7/9 (78%)	7/35 (20%)	8/9 (88.9)	0/8 (0%)	1/40 (2.5%)
Saadi J. et al. [35]; 2017	22	FIGO *: IA2 (5), IB1 (16), IB2 (1); <2 cm (21), >3 cm (1)	SSC (17), AC (5)	yes	N/A	yes	polypropylene sutures, size #0	yes	Foley catheter (7–10 days)	2-0 Vicryl suture	N/A	N/A	N/A	N/A	N/A	N/A	2/22 (9%)	2 (100%)	1/2 (50%)	1/35 (2.9%)
Saadi J.M. et al. [36]; 2015	4	FIGO **: IB1 (4)	SCC (2), AC (1), other (1)	yes	N/A	no	N/A	yes	Foley catheter (15 days)	2-0 Vicryl suture	N/A	N/A	N/A	N/A	N/A	N/A	N/A	N/A	N/A	N/A
Kim J. et al. [37]; 2010	27	FIGO **: IIA (1), IB1 (26);	SSC (20), AC (6), ASC (1);	yes	N/A	yes	polyester Mersilene tape	N/A	N/A	N/A	N/A	N/A	N/A	1/6 (16.7)	6/27 (18.75%)	3/6 (50%)	3/27 (11.1%)	1/3 (33.3)	0/1 (0%)	1/27 (3.7%)
Chen Y. et al. [38]; 2008	16	FIGO ***: IA1 (3), IA2 (7), IB1 (6)	SSC (14), AC (2)	no	N/A	yes	polypropylene sutures size #1	N/A	N/A	N/A	N/A	N/A	N/A	1/16 (6.25)	N/A	N/A	5/16 (31.3%)	2/5 (40%)	1/2 (50%)	0/16 (0%)
Ekdahl L. et al. [39]; 2021	42	FIGO *: IA1 (3), IA2 (12), IB1 (26), IIA (1)	SSC (16), AC (26)	yes	yes	yes	polypropylene sutures size #0	N/A	N/A	N/A	N/A	N/A	N/A	3/22 (14%)	28/42 (67%)	22/28 (79%)	22/42 (52.4%)	28/39 (71.8)	4/39 (10.3%)	0/42 (0%)
Park N. et al. [40]; 2009	4	FIGO *: IA2 (1), IB1 (3)	SCC (4)	no	yes	no	N/A	N/A	N/A	N/A	N/A	N/A	N/A	N/A	N/A	N/A	N/A	N/A	N/A	1/4 (25%)
Ebisawa K. et al. [41]; 2013	56	FIGO **: IA2 (4), IB1 (52)	SCC (42), AC (12), other (2)	yes	no	yes	polyester sutures size #2	N/A	N/A	N/A	N/A	N/A	N/A	7/21 (33%)	25/56 (45%)	13/25 (52%)	13/56 (23.2%)	13/21 (61.9)	10/13 (76%)	1/56 (1.8%)
Kucukmetin A. et al. [42]; 2014	11	FIGO **: 1B1 (11)	SCC (5), AC (6)	no	yes	yes	polyester sutures size #1	yes	10 days	2-0 Vicryl suture	N/A	yes	N/A	N/A	N/A	no pregnancies	N/A	N/A	N/A	0/11 (0%)
Persson J. et al. [43]; 2012	12	FIGO **: IA1 (4), IA2 (5), IB1 (3)		yes	yes	yes	polypropylene sutures size #0	no	N/A	2-0 Vicryl suture	no	N/A	N/A	N/A	5/12 (42%)	4/5 (80%)	4/12 (33.3%)	2/2 (100%)	2/2 (100%)	0/12 (0%)

N/A—not available, FIGO *—according to FIGO 2018; FIGO **—according to FIGO 2009; FIGO ***—according to FIGO1995; SCC—squamous cell carcinoma; AC—adenocarcinoma; ASC—adenosquamous cell carcinoma; T-IUD—T intrauterine device.

**Table 3:** The list of the articles and extracted data concerning vaginal technique for radical trachelectomy.

Vaginal																				
Study	Number of Cases	FIGO	Tumor Types	Technique									Median Time to Pregnancy	IVF Rate (%)	Attempt for Pregnancy (%)	Pregnancy Rate/Attempt for Pregnancy (%)	Pregnancy Rate/Number of Patients Wit RT (%)	Live Birth Rate (%)	Preterm Delivery Rate (%)	Recurrence Rate (%)
				Uterine Artery Preservation	Nerve Sparing Surgery	Abdominal Cerclage	Type of Suture (Abdominal Cerclage)	Uterine Dilatation During Cerclage Placement	Prolongation of Uterine Catheterization	Type of Utero-Vaginal Anastomosis	Extended Antibiotic Prophylaxis	Suppression of Menstruation								
Shinkai S. et al. [44]; 2022	10	FIGO *: 1A2 (2), 1B1 (8)	SSC (6), AC and ASC (4)	yes	no	yes	nylon suture (without size)	no	no	neocervix was developed by the Sturmdorf technique	no	no	N/A	any	N/A	N/A	N/A	N/A	N/A	0/10 (0%)
Plaikner A. et al. [45]; 2020	12	FIGO *: IA1 (1), IA2 (1), IB1 (7), IB2 (2), IIA1 (1)	SSC (5), ASC (6), AC (1)	yes	N/A	N/A	N/A	N/A	N/A	N/A	N/A	N/A	N/A	N/A	N/A	N/A	N/A	N/A	N/A	N/A
Meglic L. et al. [46]; 2017	15	FIGO *: A1 (5), IA2 (8), IB1 (2)	N/A about type	N/A	N/A	yes	N/A	no	no	N/A	N/A	N/A	N/A	1/15 (7.2%)	4/15 (26.7%)	3/15 (21.5%)	3/15 (21.5%)	3/3 100%	0/3 (0%)	0/15 (0%)
Takada S. et al. [47]; 2013	8	FIGO *: IA1, IA2, IB1	AC (2), SSC (6)	yes	N/A	yes	nylon suture (without size)	N/A	N/A	neocervix was developed by the Sturmdorf technique	N/A	N/A	26 months	N/A	N/A	8/7 (114)%	8/8 (100%)	8/8 (100%)	8/8 (100%)	0/8 0%
Hertel H. et al. [48]; 2006	108	FIGO *: IA1 (18), IA2 (21), IB1 (69)	SCC (75), AC (33)	yes	N/A	yes	N/A	N/A	Foley catheter (5 days)	N/A	N/A	N/A	N/A	N/A	N/A	18/108 (16.7%)	18/108 (16.7%)	15/18 (83.3%)	N/A	3/108 (2.8%)
Speiser D. et al. [49]; 2011	212	FIGO *: IA1 L1 (34), IA2 (47), IB1 (131)	SSC (154), AC (55), ASC (3)	yes	N/A	yes	N/A	N/A	Foley catheter (5 days)	N/A	N/A	N/A	N/A	4/76 (5.3%)	76/212 (35.8%)	60/76 (75.6%)	60/212 (28.3%)	45/60 (7%)	18/45 (40%)	8/212 (3.7%)
Burnett A.F. et al. [50]; 2003	18	FIGO **: IA2 (1), IB1 (20)	SSC (12), AC (9)	yes	N/A	yes	polypropylene-Prolene size 1.0; polyester Mersilene tape	N/A	N/A	N/A	N/A	N/A	N/A	N/A	N/A	3/18 (16.7%)	3/18 (16.7%)	2/3 (66.7%)	1/2 (50%)	0/18 (0%)
Dargent D. et al. [51]; 2000	47	FIGO **: IA1 (5), IA2 (13), IB1 (22), IB2 (3), IIA (1), IIB(3)	SSC (39), AC (3), ASC (4), other (1)	N/A	N/A	yes	N/A	N/A	Foley catheter (2–6 days)	N/A	N/A	N/A	N/A	N/A	N/A	25/47 (53.2%)	24/47 (51%)	13/25 (52%)	N/A	2/47 (4.3%)
Roy M. et al. [52]; 1998	30	FIGO **: IA1 (1), IA2 (7), IB1 (18), IB2 (2), IIA (2)	SSC (18), AC (12)	yes	N/A	yes	non- resorbable suture	N/A	30 days	N/A	N/A	N/A	N/A	N/A	6/30 (20%)	6/6 (100%)	6/30 (20%)	4/6 (66.7%)	2/4 (50%)	1/30 (3.33%)
Hauerberg L. et al. [53]; 2015	120	FIGO *: CIS (2), IA1 (7), IA2 (8), IB1 (96), IB2 (7)	AC (38), SSC (82)	no	N/A	yes	yes	N/A	Foley catheter (5 days)	N/A	N/A	N/A	N/A	19/72 (26.4%)	72/120 (60.0%)	55/72 (76.3%)	55/120 (45.8%)	53/77 (68.8%)	33/53 (62.3%)	6/120 (5.1%)
van der Plas R.C.J. et al. [54]; 2021	34	FIGO *: IB1 (30), IB2 (3), IB3 (1)	SSC (27), AC (6), ASC (1)	yes	N/A	yes	yes	N/A	Foley catheter (2 weeks)	N/A	N/A	N/A	N/A	6/24 (25%)	24/34 (70.6%)	15/24 (62.5%)	15/34 (44.1%)	15/20 (75%)	1/20 (5.0%)	0/34 (0%)
Bernardini M. et al. [55]; 2003	80	FIGO **: N/A	N/A	yes	N/A	yes	polyester Mersilene tape	N/A	Foley catheter (3 weeks)	N/A	N/A	N/A	11 months	3/18 (16.7%)	39/80 (48.8%)	18/39 (46.15%)	18/80 (22.5%)	18/22 (81.8%)	6/22 (27.3%)	N/A
Wang A. et al. [56]; 2019	83	FIGO *: IA1(26), IA2	SSC (78), AC (5); (11), B1 (46)	yes	N/A	yes	N/A	N/A	N/A	N/A	N/A	N/A	15 months	0/69 (0%)	69/83 (83.1%)	54/69 (84.1%)	54/83 (65.1%)	50/58 (86.2%)	8/50 (16%)	1/83 (1.2%)
Brătilă E. et al. [57]; 2015	32	FIGO **: IA1 (4), IA2 (13), IB1 (19)	SSC (34), AC (2);	yes	N/A	yes	polypropylene-Prolene size 1.0	N/A	N/A	neocervix was developed by the Sturmdorf technique	N/A	yes	2 years	1/28	28/32 (87.5%)	7/28 (25%)	7/32 (21.9%)	5/7 (71.4%)	0 (0%)	0/32 (0%)
Rizzuto I. et al. [58]; 2019	19	FIGO *: IA2 (5), IB1 (14)	SSC (12), AC (7);	no	N/A	yes	nylon suture size 1.0	yes	Foley catheter (2 days)	absorbable multifilament sutures	N/A	yes	N/A	4/19 (21.05%)	19/19 (100%)	1/19 (5%)	1/19 (5%)	N/A	N/A	4/19 (21.05%)
Covens A. et al. [59]; 1999	32	FIGO *: IB1 (31), IB2 (1)	SSC (19), AC (13)	yes	N/A	yes	polyester Mersilene tape	N/A	Foley catheter (3 weeks)	N/A	N/A	N/A	13 months	N/A	13/32 (41%)	4/13 (31%)	4/32 (12.5%)	3/5 (60%)	0/3 (0%)	1/32 (3%)
Schlaerth J. et al. [60]; 2003	10	FIGO *: IA2 (8), IB1 (2)	SSC (4), AC (5), ASC (1)	no	N/A	yes	polypropylene- Prolene size 1,0; polyester Mersilene tape	N/A	Foley catheter (few days)	N/A	N/A	N/A	N/A	N/A	N/A	4/10 (40%)	4/10 (40%)	2/4 (50%)	1/2 (50%)	0/10 (0%)
Kim M. et al. [61]; 2014	36	FIGO *: IA2 (1), IB1 (9)	SSC, AC (N/A about types)	no	N/A	yes	nylon suture (without size)	N/A	N/A	neocervix was developed by the Sturmdorf technique	N/A	no	6–80 months	N/A	N/A	9/36 (25%)	9/36 (25%)	9/10 (90%)	7/10 (70%)	0/36 (0%)
Alexander-Sefre F. et al. [62]; 2005	29	FIGO **: N/A	N/A	no	N/A	yes	nylon suture size 1.0	yes	Foley catheter (2–6 days)	absorbable multifilament sutures	N/A	N/A	6 months	N/A	N/A	N/A	N/A	N/A	N/A	N/A
Plante M. et al. [63]; 2010	125	FIGO *: IA1 (7), IA2 (29), IB1 (73), IB2 (12), IB3 (2), IIA (2)	SSC (69), AC (48), ASC (8)	no	no	yes	polypropylene- Prolene size 1.0	N/A	N/A	absorbable multifilament sutures	N/A	N/A	6–12 months after surgery patients can try to conceive	N/A	N/A	58/125 (46.4%)	58/125 (46.4%)	77/106 (72.6%)	19/77 (24.6%)	6/125 (4.8%)
Shepherd J. et al. [64]; 2001	30	FIGO **: IB1 (3)	SSC (21), AC (8), other (1)	yes	N/A	yes	nylon suture size 1.0	yes	N/A	N/A	N/A	yes	N/A	N/A	13/30 (43.3%)	14/13 (107.7%)	14/30 (46.7%)	9/14 (64.3%)	7/9 (63%)	0 (0%)
Wang Y. et al. [10]; 2021	68	FIGO *: IA1 (4), IA2 (16), IB1 (48)	SSC (49), AC (11), other (8)	yes	no	yes	N/A	N/A	Foley catheter	N/A	N/A	N/A	6 months	3/22 (13.6%)	57/68 (83.82%)	22/57 (38.6%)	22/68 (32.35%)	20/22 (90.91%)	6/20 (30%)	7 (10.3%)

N/A—not available, FIGO *—according to FIGO 2018; FIGO **—according to FIGO1995; SCC—squamous cell carcinoma; AC—adenocarcinoma; ASC—adenosquamous cell carcinoma; T-IUD—T intrauterine device.

In the case of abdominal RT, 22 studies [9,10,11,12,13,14,15,16,17,18,19,20,21,22,23,24,25,26,27,28,29], including 1712 patients, were selected. In total, 1319 patients were diagnosed with squamous cell carcinoma (SCC), 345 with cervical adenocarcinoma (AC), and 98 with other histopathological types of cancer. Concerning endoscopic RT, 14 studies [30,31,32,33,34,35,36,37,38,39,40,41,42,43] encompassing 445 patients were analyzed. SCC was diagnosed in 297 patients, while 130 had cervical AC, and 18 had other histopathological types of cancer. The selected 22 studies [10,44,45,46,47,48,49,50,51,52,53,54,55,56,57,58,59,60,61,62,63,64] referring to vaginal RT included 1158 patients; among them, 710 patients were diagnosed with SCC, 267 with cervical AC, and 28 with other histopathological types of cancer. We found no difference between the rate of SCC and cervical AC among the groups with different surgical approaches (*p* = 0.724).

### 3.2. Surgical Technique for Abdominal Radical Trachelectomy

The uterine artery was preserved in 12 (12/22; 54.55%) studies [9,10,12,13,15,19,20,21,25,27,28], while it was resected during lateral parametrectomy in 7 (31.82%) studies [11,14,16,17,18,23,24]. Three (13.64%) studies did not report uterine artery preservation or resection [22,26,29]. Autonomic nerve-sparing surgery was performed in 8 (36.36%) studies [9,13,15,21,25,27,28,29], while 5 (22.73%) reported traditional parametrectomy without nerve-sparing techniques [9,10,14,16,24]. 9 (40.91%) studies did not report the method of the surgery regarding the preservation of the pelvic autonomic nervous system [11,12,17,18,19,20,22,23,26]. In 16 studies (72.73%), the permanent abdominal cerclage was used to decrease the risk of premature delivery [10,12,13,14,15,16,17,19,20,21,22,24,25,26,27,28]. However, in 3 (13.64%) studies [11,18,23], the abdominal cerclage was not performed, and another 3 (13.64%) studies did not provide data in this respect [9,29]. A variety of sutures and non-absorbable meshes were used as abdominal cerclage. The most common were polyester suture size #0 (4 studies) [13,22,25,28], suture made of expanded polytetrafluoroethylene (ePTFE) GoreTex (2 studies) [24,26], and polyester Mersilene tape (2 studies) [14,19].

To prevent cervical stenosis due to the cerclage, cervical catheterization may be used. However, only 10 (45.45%) [10,11,12,13,14,15,19,20,24,25] studies reported the use of cervical catheterization and provided data on the used catheter (in the majority of cases, the Foley catheter) and the prolongation of the catheterization. The median duration of catheterization was 24.5 days (range 7–56 days). Foley catheter use was reported in 18.18% (N = 4) of studies [10,12,13,15]. In three cases, the T intrauterine device was used [14,19,24]. In eight studies (38.1%), the authors used interrupted, absorbable sutures for utero-vaginal anastomosis after radical abdominal trachelectomy [11,14,15,16,18,19,20,24]. In the rest of the studies, the exact type of utero-vaginal anastomosis was not described. Extended antibiotic treatment over prophylactic antibiotic therapy was only used in 1 (4.76%) study [15]. Suppression of menstruation was not reported in any of the analyzed studies.

### 3.3. Surgical Technique for Endoscopic Radical Trachelectomy

In 10/14 (71.43%) studies, the uterine artery was preserved, while in four studies (23.57%), it was resected [30,38,40,42]. Autonomic nerve-sparing surgery was performed in half of the studies (N = 7/14, 50%), and only 2/14 (14.29%) used a non-sparing method [31,41]. The remaining articles (N = 5, 35.71%) did not mention their approach to sparing nerves [30,35,36,37,38]. In 10 out of 14 publications (71.43%), the abdominal cerclage was placed to prevent premature delivery [30,31,34,35,37,38,39,41,42,43], while in 3/14 (21.43%), it was not applied [33,36,40]. One study did not mention applying abdominal cerclage [32]. To perform abdominal cerclage, many different materials were used. The most commonly used (6; 42.86%) were non-absorbable, monofilament, polypropylene sutures, size #0 and size #1 [31,34,35,38,39,43]. Some studies applied polyester sutures size #1 and size #2 [41,42]. Moreover, polyester Mersilene tape was used in one study [37]. In some of the reviewed articles (N = 535.71%), intrauterine catheters to prevent cervical stenosis were used [32,33,35,36,42]. The prolongation of catheterization differed from 7 days up to 6 months, with a median duration of 15 days. Foley catheter use was reported in 21.43% (N = 3) of studies [35,36,42]. In eleven (78.57%) publications, the authors described their technique for the utero-vaginal anastomosis; in four studies (28.57%), the 2-0 Vicryl suture was used [35,36,42,43] while in seven studies there is no information about using suture type. Extended postoperative antibiotic prophylaxis was only applied in one study (7.14%) [30]. Similarly, suppression of menstruation postoperatively was only reported in one (7.14%) study [42].

### 3.4. Surgical Technique for Vaginal Radical Trachelectomy

The uterine artery was preserved in 14/22 (63.64%) studies [10,44,45,47,48,49,50,52,54,55,56,57,59,64] while it was resected in 6/22 (27.27%) studies [53,58,60,61,62,63]. Two (9.09%) studies did not provide data on uterine artery preservation or resection [46,51]. In the majority of the studies (N = 21/22; 95.45%), a permanent cervical cerclage was used to decrease the risk of premature delivery [10,44,46,47,48,49,50,51,52,53,54,55,56,57,58,59,60,61,62,63,64]. However, in the case of one study (4.55%), the cerclage was not reported [45]. A variety of different sutures or non-absorbable meshes were used as cervical cerclage. A monofilament non-absorbable nylon suture, mostly size 1.0, was most commonly used (N = 6, 27.3%) [44,47,58,61,62,64], followed by polypropylene- Prolene, size 1.0 (4 studies; 18.18%) [50,57,60,63] and polyester fibers-Mersilene tape (4 studies; 18.18%) [50,55,59,60].

The use of uterine catheterization was reported in 12 among 22 studies (54.55%) [10,48,49,51,52,53,54,55,58,59,60,62]. The median duration of uterine catheterization was 5 days, ranging from 2 to 30 days [48,49,51,52,53,54,55,58,59,60,62]. Foley catheter use was reported in all of the studies with reported uterine catheterization (N = 12, 54.55%). In three studies (13.64%), absorbable multifilament sutures were used for utero-vaginal anastomosis after vaginal RT [58,62,63]. After the cervix was removed, the neocervix was developed by the Sturmdorf technique in 4/22 studies (20%) [44,47,57,61]. None of the studies applied extended antibiotic prophylaxis. Suppression of menstruation with oral contraception for 6–12 months was reported in 3 studies (13.64%) [57,58,64].

### 3.5. Surgical Techniques in Various Surgical Approaches for Radical Trachelectomy

**Figure 2:**
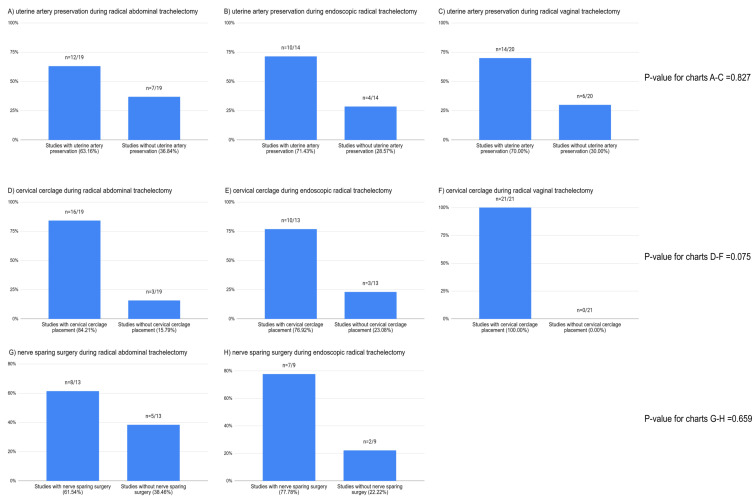
Studies concerning different approaches for and surgical steps during radical trachelectomy. The *p*-value was calculated using a contingency table. Articles with no data on the management of uterine arteries and autonomic nerves were not included in the calculation.

We have summarized the number of studies that used uterine artery preservation, cervical cerclage, and nerve-sparing techniques for RT in Figure 2. In summary, when all of the surgical approaches were analyzed together, the uterine artery preservation, the cervical cerclage, and the nerve-sparing technique were used in 62.07%, 81.03%, and 25.86% of the studies, respectively. There were no significant differences in the use of the above-mentioned techniques across different surgical approaches for RT (Figure 2, Supplementary Table S2).

**Figure 3:**
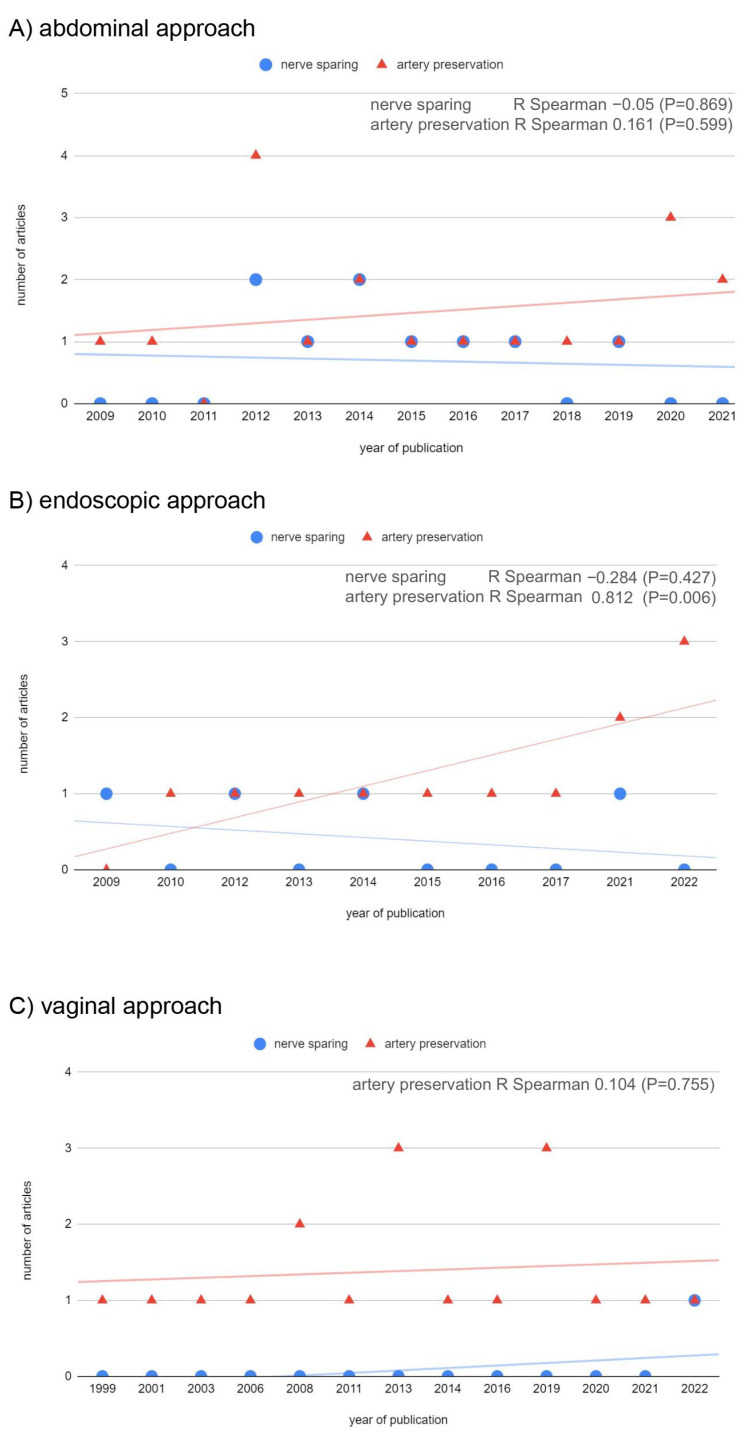
The trend in the use of uterine-sparing and nerve-sparing surgery for radical trachelectomy. The figure presents the trends in publishing studies concerning radical trachelectomy using nerve-sparing techniques and uterine artery preservation.

We have evaluated the use of nerve-sparing and uterine-sparing procedures over the years. The data available for this evaluation were found in the studies published between 1999 and 2022. We observed an increasing trend in uterine-artery sparing RT in the endoscopic approach (R Spearman = 0.812, *p* = 0.006) and when all studies were evaluated together (R Spearman = 0.560, *p* = 0.016). The trend in the case of abdominal and vaginal approaches was not statistically significant (Figure 3). In the case of the nerve-sparing technique, only abdominal and endoscopic approaches were evaluated, but no change in the frequency of nerve-sparing technique application in recent years was noted (Figure 3).

We did find, however, significant differences in the use of the nerve-sparing techniques according to the stage of the disease. For both abdominal and endoscopic approaches, the nerve-sparing technique was more commonly used in the FIGO IA stage when compared to the FIGO IB stage. Similarly, uterine artery-sparing surgery was more common among early-stage CC treated with the vaginal approach. Nevertheless, the differences in sparing the uterine artery were insignificant in the case of abdominal and endoscopic approaches. (Table 4).

### 3.6. The Relationship Between Surgical Techniques During RT and Oncological and Obstetrical Outcomes

**Figure 4:**
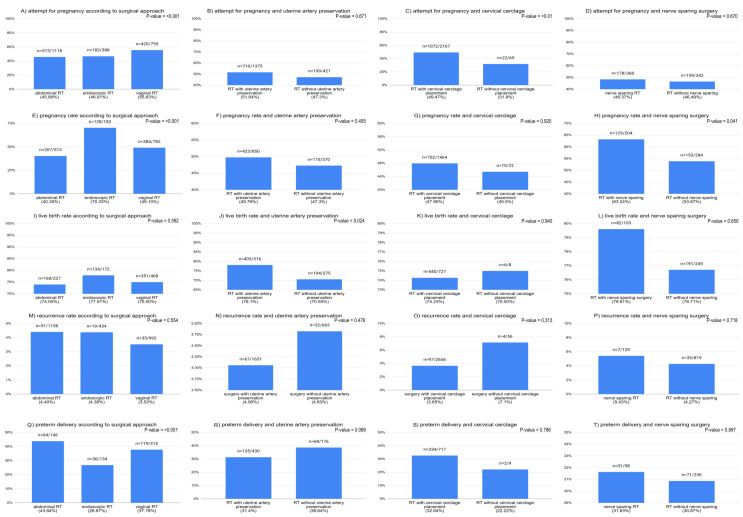
The relationship between surgical techniques during radical trachelectomy and oncological and obstetrical outcomes. The ‘attempt for pregnancy rate’ was defined as the rate of patients who declared trying for pregnancy. The ‘pregnancy rate’ was defined as the rate of patients who became pregnant. The ‘live birth rate’ was defined as the rate of live births after 24 weeks of pregnancy among all patients who have had RT. The ‘pregnancy rate among attempt’, which was defined as the pregnancy rate among patients who attempted to become pregnant. The ‘preterm delivery rate’ was calculated as the rate of deliveries before 37 weeks of gestation among all live births.

An increased rate of attempts for pregnancy after vaginal RT than after abdominal and endoscopic RT was observed. The endoscopic approach for RT was associated with a significantly higher pregnancy rate when compared to vaginal and abdominal approaches. Similarly, the rate of preterm deliveries was lower in the endoscopic approach. However, there were no differences in the live birth rate between the analyzed surgical approaches. There were also no differences in the recurrence rate for cancer between the different approaches for RT. The results are summarized in Figure 4 and Supplementary Table S3.

A significantly higher live birth rate was observed among patients who had uterine artery preservation when compared to patients who had RT with uterine artery dissection. However, uterine artery management was not associated with any other outcome (attempt for pregnancy, pregnancy rate, recurrence rate, and preterm delivery). Patients who had abdominal cerclage placed during RT were found to have a significantly higher attempt for pregnancy rate when compared to patients who had no abdominal cerclage. Again, no association with the pregnancy rate, live birth rate, recurrence rate and, surprisingly, preterm delivery rate was observed. Finally, nerve-sparing RT was associated with a higher pregnancy rate when compared to patients who had traditional parametrial resection. There were no differences in the attempt for pregnancy, live birth rate, and recurrence rate regarding the preservation of the pelvic autonomic nervous system during RT. The results are summarized in Figure 4.

### 3.7. Adverse Events

We found a higher infection rate in the abdominal RT cohort when compared to endoscopic and vaginal RT. The vaginal approach for RT was characterized by a lower rate of secondary infertility and a higher rate of postoperative vaginal bleeding and lymphatic complications. There were no differences in the rate of urinary tract adverse events or cervical stenosis between the analyzed surgical approaches for RT. The results are summarized in Table 5. The adverse events mentioned in the analyzed articles are included in Supplementary Table S1.

## 4. Discussion

This systematic review attempted to summarize the surgical techniques used during RT due to CC. Furthermore, certain surgical procedures were compared and analyzed with regard to the obstetrical outcomes and oncological safety to help clinicians choose the optimal surgical technique and adequately counsel the patient when RT is considered. We found similar oncological outcomes after different surgical approaches to RT. The MIS approach was associated with an increased pregnancy rate and lower preterm birth rate. We observed that uterine artery-sparing and nerve-sparing techniques increased live birth and pregnancy rates, respectively. Permanent cervical cerclage is widely used among different authors and the Foley catheter was the most frequently used to prevent cervical stenosis following RT.

We have identified a similar number (n = 22) of studies concerning abdominal and vaginal approaches for RT. However, the studies evaluating the abdominal approach included half as many patients as the vaginal approach. The endoscopic approach was reported in a smaller number of studies and included a lower number of patients. After the publication of the Laparoscopic Approach for Cervical Cancer (LACC) trial in 2018, a decline in the use of minimally invasive techniques for radical hysterectomy was observed [65]. The main concern related to endoscopic surgery for CC pertains oncological safety. Nevertheless, in our study, we found similar recurrence rates for all surgical approaches (3.53–4.4%), which corresponds to the results of other studies [66,67,68,69], including meta-analyses and systematic reviews. In the meta-analysis by Han et al., the authors observed no significant difference in the overall survival and recurrence rate between the MIS and abdominal RT groups [70]. The pooled recurrence rates in this meta-analysis were 5.5% and 4.4% for the MIS and abdominal surgery groups, respectively [70]. Another systematic review summarized data from 53 studies on RT and reported similar oncological outcomes for all three surgical approaches: the recurrence rates for vaginal vs. abdominal vs. MIS were 4% vs. 3.9% vs. 4.2%, respectively [69]. These studies support the safety of the endoscopic approach for RT. On the contrary, the LACC trial showed better disease-free survival and overall survival for CC patients treated with laparotomy than for MIS patients. Although the LACC trial assessed radical hysterectomy and was not powered enough to evaluate the oncological outcomes in small tumors, also in the tumors less than 2, the recurrence rate in MIS was much higher when compared to patients treated with laparotomy [65,71] we believe that MIS in RT should be performed cautiously and only in patients selected by an experienced team. Furthermore, avoiding the uterine manipulator and using maneuvers to prevent tumor spread at the time of colpotomy in minimally invasive surgery is encouraged [72].

In our study, significantly higher pregnancy rates were observed after endoscopic rather than after vaginal and abdominal RT. Similar findings are reported in the review from 2016 [73]: pregnancy rates after abdominal vs. vaginal vs. minimally-invasive RT were 44% vs. 57% vs. 65%, respectively. However, data in the literature are inconsistent on this matter. In another small dataset, the pregnancy rates were 31.3% and 51.5% in the MIS and abdominal RT groups, respectively, favoring open surgery [70]. Another systematic review reported the highest pregnancy rates in the vaginal RT group and the lowest in the MIS group (49.4% vs. 43.2% vs. 36.2% for the vaginal vs. abdominal vs. MIS RT, respectively) [69]. An extensive review comprising over 6000 patients undergoing fertility-sparing treatment due to CC reported the lowest pregnancy rates in the abdominal RT group (36%) and the highest in the robotic RT group (77%). Pregnancy rates in the vaginal and laparoscopic groups were 58.7% and 50%, respectively [68]. Lower pregnancy rates after the abdominal approach can be attributable to various reasons. Firstly, abdominal surgery may cause adhesions that reduce fertility. Another reason for decreased fertility might be the generally higher rate of post-surgical complications after laparotomy, especially peritonitis.

In our systematic review, we found no differences in live birth rates between abdominal, vaginal, and endoscopic approaches for RT. Similar results were found in the study by Bentivegna et al. and Morice et al., where the live birth rate did not depend on the surgical approach [69,74]. Nevertheless, the data in the literature on this topic are inconsistent. In the systematic review by Kuznicki et al., the author found a lower live birth rate (44%) in the abdominal RT cohort when compared to vaginal and MIS RT (65% and 57.1%, respectively) [69]. On the other hand, in the meta-analysis by Lv et al., the third-trimester delivery rate was higher in the abdominal approach compared to MIS [67].

Pregnancy after RT is typically considered high risk, with preterm delivery being one of the primary concerns. The main reasons for pregnancy complications after RT are associated with a shortened cervix: cervical insufficiency, preterm premature rupture of membranes (pPROM), and chorioamnionitis [74,75,76]. In our study, preterm delivery rates ranged from 27 to 38% according to the surgical approach, favoring the MIS. In a review by Bentivegna et al., the reported prematurity rate ranged from 39 to 57% and was the lowest for the vaginal approach [73]. Similarly to our findings, in an extensive dataset reported by Morice et al., the prematurity rates for all surgical approaches were comparable and slightly in favor of MIS (prematurity rates for vaginal/abdominal/laparoscopic/robotic RT were 30%/29%/24.6%/23.5%, respectively) [68].

The uterine artery is the main artery responsible for the vascular supply of the uterus. At the level of the uterine isthmus, the uterine artery divides into ascending and descending branches. Preserving the uterine artery with its ascending branch during RT is assumed to guarantee adequate uterine vascularization; therefore, better obstetrical outcomes may be expected. On the other hand, the uterine artery is part of the lateral parametrium (or vascular mesometrium); therefore, its preservation may hamper the oncological safety of the surgery. There is limited data regarding the influence of the uterine artery-sparing technique on pregnancy rate, neonatal outcome, and long-term oncological safety. In the study by Escobar et al., the authors compared the vascularization of the uterine fundus using indocyanine green (ICG) after uterine artery-sparing and non-sparing RT [77]. There were no differences in mean ICG fluorescence nor in the pregnancy rate between the groups, although the strength of these findings is limited as there were only seven pregnancies [77]. In our study there were also no differences in the pregnancy rate; however, we did observe a statistically significant increase in the live birth rate in the uterine artery-sparing group. This may be explained by theoretically better uterine perfusion when a uterine artery is preserved. Taking into account similar recurrence rates in both groups, it seems that the preservation of the uterine artery during RT should be the preferred option. In general, this trend can be noticed in the literature as we found that the use of the uterine artery-sparing technique has been increasing in recent years.

The history of nerve-sparing surgery in radical hysterectomy is related to the pioneer works of Professor Okabayashi from Kyoto University in Japan [77]. This technique was developed and expanded in Europe by Professor Hockel from Leipzig University in Germany, who described Total MesoMetrial Resection (TMMR) for cervical cancer [78]. Nerve-sparing radical hysterectomy is associated with less urinary, colorectal, and sexual dysfunction [79]. Although previous studies suggested similar oncological outcomes of nerve-sparing radical hysterectomy compared to traditional radical hysterectomy, recent analysis by Bizzarri et al. showed that in the group of 2–4 cm tumors, more radical and traditional surgery was associated with improved survival [79,80]. However, in tumors less than 2 cm, no survival difference was found with more radical hysterectomy, and therefore, the nerve-sparing technique in this group of patients seems safe [80]. Although the nerve-sparing techniques in RT have been increasingly used in recent years, this trend was not statistically significant. Our systematic review observed no differences in the attempt for pregnancy, live birth rate, recurrence rate, and preterm delivery rate when the nerve-sparing technique was compared to traditional surgery, during which the autonomic nervous system was not explicitly preserved. However, we observed an increased pregnancy rate in the nerve-sparing RT group. This may be linked to less sexual dysfunction in patients after nerve-sparing surgery [79]. Recently, a novel technique of nerve-sparing RT with the preservation of uterine branches was introduced. The method seems feasible; however, its impact on future fertility and oncological outcomes remains to be determined [81].

Patients after RT have significantly shortened cervix, which can result in cervical insufficiency during future pregnancies. Routine placement of a cervical cerclage can reduce the risk of preterm delivery. In the study by Mathevet et al., cervical cerclage placed after vaginal RT reduced the risk of fetal loss from 50% to 22% [82]. Our systematic review shows that most surgeons routinely place cervical cerclage during RT, regardless of the surgical approach. It was used in 72.73%, 71.43%, and 95.45% of studies reporting abdominal, MIS and vaginal approach for RT, respectively. Various non-absorbable sutures or tapes can be used as cervical cerclage. In the case of repeated early pregnancy loss due to cervical insufficiency in patients not treated with RT and abdominal cerclage, using nonabsorbable tape is one of the most commonly used options [83]. In the case of RT, there is usually not enough space for a 5 mm tape; therefore, a suture is more feasible and was used by a majority of authors in our series. Our systematic review shows that the sutures most commonly used for a cerclage during RT are nonabsorbable, monofilament polypropylene, polyester, or nylon sutures sizes 0 and 1.

One of the most common adverse events related to cervical cerclage placement is cervical stenosis that can cause obstruction of menstrual outflow and infertility. In our study, the incidence of cervical stenosis did not depend on the surgical approach. Similar results were found in the meta-analysis by Han et al. [70]. In a systematic review by Li et al. [84] comprising data on 1547 patients that underwent RT, the rates of postoperative cervical stenosis for abdominal, vaginal, laparoscopic, and robotic approaches were 11.0%, 8.1%, 9.3%, and 0%, respectively, whereas in this our study these rates were 8.79%, 8.77%, 11.04% for the abdominal, endoscopic and, respectively. The incidence of cervical stenosis was associated with cerclage placement (8.6% after cerclage placement vs. 3.0% without cerclage) but not with the type of the suture used—it occurred in 10.7% vs. 8.1% in the non-braided vs. braided type of suture used for the cerclage, and the difference was not statistically significant [84]. Furthermore, anti-stenosis tools, such as catheters or intrauterine devices, were shown to decrease the incidence of cervical stenosis 3-fold [84]. In the systematic review by Li et al. [84], anti-stenosis tools were used in 27.4% of the RT cases. In our study, this rate was much higher: 45.45%, 35.71, and 54.55% for the abdominal, endoscopic, and vaginal approach for RT, respectively. A Foley catheter was the most frequently used device for cervical canal catheterization—its use was reported in 18–36% of the analyzed studies Similarly, in the paper by Li et al. [84], the most commonly used anti-stenosis tool was a Foley catheter (68.4%), followed by an IUD (29%). The duration of catheterization varied between the studies with the median duration of approximately 1–2 weeks.

Prolonged surgery, opening of the vagina, as well as placement of artificial materials like cervical cerclage or uterine catheter may increase the risk of postoperative infection. This is especially relevant for young women with a pregnancy desire. However, extended antibiotic prophylaxis over traditional single-dose prophylaxis was routinely administered only in two studies concerning abdominal RT, two studies on endoscopic RT, and none of the studies on vaginal RT.

One of the last steps during RT is utero-vaginal reanastomosis. In the vaginal approach, the most commonly reported technique for neocervix formation was the Sturmdorf technique, in which the vaginal mucosa is used to cover the exposed cervix. In the case of abdominal and minimally invasive approaches, the utero-vaginal reanastomosis is more challenging. In most of the studies, interrupted absorbable sutures were used to reconnect the uterus with the vaginal cuff. Although the exact suturing technique was rarely provided, restoring anatomical junction is possible with the “cuff-sleeve” method of the reanastomisis. In this technique, the vagina is sutured around the cervical stump, and finally, wraps around the cervix like a sleeve. This method is well described by Xu et al. [33].

As RT is a surgery designed for fertility preservation, the patients are expected to regain regular menstruations. Early menstruation following RT may raise concerns regarding proper healing. However, suppression of menstruation after RT was very uncommon.

Although the general rate of surgery-related adverse events after RT was low, several differences between analyzed RT approaches could be noticed. Not surprisingly, we observed a higher rate of infection complications in the abdominal RT group [85]. Secondary infertility is a common problem after RT, and in vitro fertilization is used in up to 64% of patients [28]. We found a lower rate of secondary infertility, in the group with a vaginal approach; this may be attributed to a lower adhesion formation after vaginal surgery. We also observed a higher rate of vaginal bleeding after vaginal RT; similar findings were reported by Einstein et al. [86]. We found that lymphatic complications occurred more often after vaginal RT in our cohort. However, a high rate of lymphatic complications in vaginal RT was due to a single study by Hauerberg et al. [53], who reported lymphoedema in as many as 40% of patients—a rate much higher than other authors. These discrepancies could be related to various diagnostic criteria for ‘lymphatic complications’ used by different authors. In most studies, the authors did not provide their diagnostic criteria for lymphatic complications, and in some, only symptomatic lymphoedema or lymphoedema requiring drainage was included.

In the meta-analysis by Lv et al., the authors found no statistically significant difference in postoperative complications, other than increased blood loss in the abdominal RT group, between laparotomy and MIS groups [67]. Similarly, in the meta-analysis by Han et al., no significant difference was found in urinary tract complication, cerclage erosion, or cervical stenosis between patients treated with MIS and abdominal approach [70].

However, the comparison of surgically related adverse events in systematic reviews and meta-analyses has a significant bias, as different authors used distinct criteria to diagnose adverse events. Therefore, the results of our study should be interpreted with caution in this respect. 

Our study has several strengths and limitations. In this systematic review, we have evaluated the techniques used during RT in detail. We aimed to focus on each RT step and summarize their usage among different authors. We think that our work may serve as a guide for clinicians when RT is considered. We also assessed the clinical performance of several steps, like uterine artery preservation and nerve-sparing technique during RT. To the best of our knowledge, this is the first study that evaluates these two techniques in systematic review. The main disadvantage of our systematic review was the exclusion of many studies that used a mixed RT approach where the data extraction for comparison of the surgical techniques was impossible. Furthermore, some of the studies included patients after neoadjuvant chemotherapy. They were also included whenever the exclusion of these patients was not possible. Another limitation is that the data were obtained from small studies, as RT is infrequently performed. Another limitation is related to the incomplete or unclear definitions, such as adverse surgical events, attempts to conceive, and nerve-sparing techniques. Therefore, bias may be due to differences in data reporting in the original studies.

## 5. Conclusions

This systematic review analyzed the impact of various surgical approaches and surgical techniques in RT, fertility-sparing surgery in early-stage cervical cancer, on reproductive and oncological outcomes. We found similar oncological outcomes after different surgical approaches to RT. Although the MIS approach was associated with an increased pregnancy rate and lower preterm birth rate, the safety of this method should be validated in prospective trials. Otherwise, this technique should be used only in experienced teams due to the possible increased risk of recurrence. Uterine artery-sparing and nerve-sparing techniques are encouraging; they increase live birth and pregnancy rates. Permanent cervical cerclage is widely used among different authors. Among anti-stenosis tools, the Foley catheter is the most frequently applied.

## Figures and Tables

**Table 4:** Uterine artery- and nerve-sparing technique according to the stage of cervical cancer and type of surgical approach for radical trachelectomy.

	Nerve Sparing Technique	Traditional Surgery	*p*-Value
FIGO stage	Abdominal approach		
IA	78 (100%)	0 (0%)	<0.001
IB	130 (43.92%)	166 (56.08%)	
	Endoscopic approach		
IA	38 (88.37%)	5 (11.63%)	0.002
IB	100 (64.52%)	55 (35.48%)	
	**Uterine Artery Preservation** **(No of Cases)**	**Uterine Artery Dissection** **(No of Cases)**	***p*-Value**
	Abdominal approach		
IA	112 (80%)	28 (20%)	0.210
IB	377 (70.86%)	155 (29.14%)	
	Endoscopic approach		
IA	47 (97.92%)	1 (2.08%)	0.201
IB	159 (91.91%)	14 (8.09%)	
	Vaginal approach		
IA	155 (84.24%)	29 (15.76%)	<0.001
IB	388 (75.19%)	128 (24.81%)	

**Table 5:** Surgery-related adverse events depending on the approach for RT.

Adverse Events		Abdominal Number of Events/ Number of Patients	Endoscopic Number of Events/ Number of Patients	Vaginal Number of Events/ Number of Patients	*p*-Value
Cervical stenosis	Yes	97/1103	35/399	53/480	0.355
		(8.79%)	(8.77%)	(11.04%)	
	No	1006/1103	364/399	427/480	
		(91.21%)	(91.23%)	(88.96%)	
Infections complications	Yes	55/494	19/331	25/441	<0.001
		(11.13%)	(5.74%)	(5.67%)	
	No	439/494	312/331	416/441	
		(88.87%)	(94.26%)	(94.33%)	
Lymphatic complications	Yes	45/494	32/331	73/446	<0.001
		(9.11%)	(9.67%)	(16.37%)	
	No	449/494	229/331	373/446	
		(90.89%)	(69.18%)	(83.63%)	
Secondary infertility (fallopian tube obstruction, amenorrhea)	Yes	38/854	26/331	5/326	<0.001
		(4.45%)	(7.85%)	(1.53%)	
	No	816/854	305/331	321/326	
		(95.55%)	(92.15%)	(98.47%)	
Urological adverse events	Yes	35/494	12/331	21/409	0.09
		(7.09%)	(3.63%)	(5.13%)	
	No	459/494	319/331	388/409	
		(92.91%)	(96.37%)	(94.87%)	
Vaginal bleeding	Yes	5/494	8/331	31/326	<0.001
		(1.01%)	(2.42%)	(9.51%)	
	No	489/494	323/331	295/326	
		(98.99%)	(97.58%)	(90.49%)	

Infectious complications in infectious complications included the following: wound infection, febrile morbidity, vaginal discharge, pelvic peritonitis, and formation of pelvic abscess. Lymphatic complications included lymphoceles, lower limb edema, pelvic lymph seroma, and temporary vulvar edema) Secondary infertility included fallopian tube obstruction, Asherman syndrome, and amenorrhea. Urological adverse events included urinary bladder dysfunction, stress incontinence, urinary retention, lower urinary tract infection, bladder injury, and fistula formation.

## Data Availability

The data presented in this study are available in this article and Supplementary Materials.

## Supplementary Materials

The following supporting information can be downloaded at: https://www.mdpi.com/article/10.3390/cancers17060985/s1, Table S1: Adverse events mentioned in analyzed articles; Table S2: Number of studies concerning radical trachelectomy; Table S3: The relationship between the surgical technique during radical trachelectomy and oncological and obstetrical outcomes. References [9,10,11,12,13,14,15,16,17,18,19,20,21,22,23,24,25,26,27,28,29,30,31,32,33,34,35,36,37,38,39,40,41,42,43,44,45,46,47,48,49,50,51,52,53,54,55,56,57,58,59,60,61,62,63,64] are cited in the Supplementary Materials.
