# Supplementary material for: Surgical Techniques for Radical Trachelectomy
Source: Cancers (Basel). 2025 Mar 14;17(6):985. doi: 10.3390/cancers17060985 (PMC11940279; doi:10.3390/cancers17060985)
Supplement: Supplementary file 1 [file cancers-17-00985-s001.zip › cancers-3456652-supplementary.pdf]

Supplementary Table S1. Adverse events mentioned in analyzed articles.

Study	Surgery adverse events
**ABDOMINAL**	
Kiss SL [1] et al.; 2021	urinary bladder dysfunction (6); pelvic lymphoceles (2); infection (3); cervical stenosis (1); amenorrhea (2)
Wang Y. et al. [2]; 2020	cervical stenosis (2)
Mabuchi S. et al. [3]; 2017	lymphocyst (1)
Li X. et al. [4]; 2016	ureteral injury (1); intestinal injury (1); febrile morbidity (18); bladder hypotonia (5); incisions infection (1); lymphocyst (5); lower extremity edema (4); vaginal discharge (10); vaginal bleeding (5); cervical stenosis (8)
van Gent M.D. et al. [5]; 2014	N/A
Lintner B. et al. [6]; 2013	cervical stenosis (1); urethral injury (1); ureteral stenosis (1); urinary retention (5); constipation (4)
Muraji M. et al.[7] ; type III; 2012	lymphocyst (1); cervical stenosis (1); amenorrhea (1)
Muraji M. et al.[7], type II with nerve- sparing; 2012	lymphoceles (3); cervical stenosis (1); amenorrhea (1)
Wethington S.L. et al.[8]; 2012	cervical stenosis (12); lymphoceles (4); postoperative ileus (4; cerclage erosion (2)
Karateke A. et al. [9]; 2012	late uretrotubal abscess (1); left leg lymphedema (1); lymphocele (1)
Li J. et al. [10]; 2011	cervical stenosis (5); lymphoceles (2); vesical dysfunction (1)
Nishio H. et al.[11]; 2009	cervical stenosis (1); amenorrhea (5); laparotomy for secondary hemorrhage (1); postsurgical infection (15); lymphoceles (9)
Okugawa K. et al. [12]; 2019	cervical stenosis (36)
Kim C.H. et al. [13]; 2012	cervical stenosis (10)
Căpîlna M.E. et al. [14]; 2014	bladder dysfunction (8); prolonged constipation (6); lymphoceles (2); amenorrhea (3); peritonitis (1); cervical stenosis (1)
Li X. et al. [15]; 2019	N/A
Nakajima T. et al. [16]; 2020	cervical stenosis (12)
Li X. et al. [17]; 2020	cervical stenosis (26); fallopian tube obstruction (22)
Tamauchi S. et al. [18]; 2016	cervical stenosis (8); cervicitis (2); lymphoceles (3); amenorrhea (3); dysuria (1)
Nishio H. et al.[19]; 2013	cervical stenosis (4)
Tokunaga H. et al.[20]; 2014	urethral injury (1); ileus (1); pelvic lymphocyst (4)
Wang Y. et al. [21]; 2021	incision infection (2); poor wound healing (3); stress incontinence (7; ileus (8); lymphoceles (5)
**ENDOSCOPIC**	
Johansen G. et al. [22]; 2016	cerclage erosions (4); cervical stenosis (1); fistula (1)
Ekdahl L et al. [23]; 2021	cervical stenosis (18); cerclage erosions (3); bladder injury (1); compartment syndrome of the leg (1); pelvic lymph seroma (4); pelvic hematoma (2); bowel obstruction (1); vaginal bleeding (8); voiding problems (4); vesico-vaginal fistula (1)
Xu M. et al.[24]; 2022	pelvic lymphoceles (3); fever (3)
Xu M et al. [25]; 2022	fever (6); urinary tract infection (3); lymphoceles (9)
Kanao H. et al.[26,27]; 2021	internal hernia of the small bowel (1); peritonitis (1)

Saadi J. et al. [27]; 2017	cervical stenosis (1), subcutaneous emphysema (2); lymphoceles (2); urinary tract infection (2); pelvic hematoma (1); abdominal abscess (1); Asherman syndrome (1)
Saadi J.M. et al. [28,29]; 2015	left vulvar edema (1)
Kim J. et al. [29]; 2010	no significant perioperative and postoperative complications
Chen Y et al. [30]; 2008	amenorrhea (1); cervical stenosis (1)
Ekdahl L et al. [31]; 2021	sex causing physical pain (16); lymphedema (15); dyspareunia (21); cervical stenosis (3)
Park N et al. [32]; 2009	N/A
Ebisawa K et al. [33]; 2013	cervical stenosis (5)
Kucukmetin A et al. [34]; 2014	cervical stenosis (1); fistula (1); infection (1)
Persson J et al. [35]; 2012	cervical stenosis (3)
**VAGINAL**	
Shinkai S et al. [36]; 2022	N/A
Plaikner A et al. [37]; 2020	fever (1); lower urinary tract infection (1)
Meglic L et al.[38]; 2017	N/A
Takada S et al. [39]; 2013	N/A
Hertel H et al. [40]; 2006	bleeding (1); embolism of the external iliac artery (1); lymphocele (1); lower limb edema (1); small bowel obstruction (1); neuropathy (4); cervical stenosis (8)
Speiser D et al. [41]; 2011	N/A
Burnett AF et al. [42]; 2003	transient neuropathy (3); lower abdominal ecchymosis (1); hematuria (1); lymphocele (1)
Dargent D et al. [43]; 2000	N/A
Roy M et al. [44]; 1998	; iatrogenic cystotomy (1), temporary vulvar edema (3); lymphoceles (3); cervical stenosis (1); vaginal bleeding (2)
Hauerberg L et al. [45]; 2015	lymphedema (48); cervical stenosis (28)
van der Plas RCJ et al. [46]; 2021	cervical stenosis (3)
Bernardini M et al. [47]; 2003	N/A
Wang A et al. [48]; 2019	infection (1); urinary retention (3); intestinal obstruction (2)
Brătilă E et al. [49]; 2015	cystotomy (2); ureter injury (1); lymphoceles (2); vulvar edema (1); suprapubic edema (2); dysmenorrhea (4); vaginal bleeding (9); abnormal uterine bleeding (2)
Rizzuto I et al. [50]; 2019	vesico-vaginal fistula (1); dyspareunia (1); vaginal infection (1); hematometra (1)
Covens A et al. [51]; 1999	cystotomies (6); trocar-induced external iliac arteriotomy (1); postoperative infection (3)
Schlaerth J et al. [52]; 2003	cystotomies (2); pelvic hematoma (1); cervical stenosis (2)
Kim M et al. [53]; 2014	N/A
Alexander-Sefre F et al. [54]; 2005	lymphodema (3); lymphoceles (3); vaginal granulation tissue (10); chronic pelvic pain (10); dyspareunia (20); vaginal discharge (14); neuropathy (14); cervical stenosis (10); erosions (4)
Plante M et al. [55]; 2010	N/A
Shepherd J et al. [56]; 2001	amenorrhea (1)
Wang Y. et al. [21]; 2021	incision infection (1); poor wound healing (1); stress incontinence (4); ileus (4); lymphoceles (5)

Supplementary Table S2. Number of studies concerning radical trachelectomy.

	Yes No of studies (%)	No No of studies (%)	No data No of studies (%)	Total	P-value
**Uterine artery preservation**					
Abdominal	12 (54.55%)	7 (31.82%)	3 (13.63%)	22	0.827
Endoscopic	10 (71.43%)	4 (28.57%)	0 (0.00%)	14	
Vaginal	14 (63.63%)	6 (27.27%)	2 (9.1%)	22	
**Abdominal cerclage**					
Abdominal	16 (72.74%)	3 (13.63%)	3 (13.63%)	22	0.075
Endoscopic	10 (71.43%)	3 (21.43%)	1 (7.14%)	14	
Vaginal	21 (95.45%)	0 (0.00%)	1 (4.55%)	22	
**Nerve sparing surgery**					
Abdominal	8 (36.36%)	5 (22.73%)	9 (40.91%)	22	0.659
Endoscopic	7 (50.00%)	2 (14.29%)	5 (35.71%)	14	

The P-value was calculated using a contingence table. Articles with no data on the management of uterine artery and autonomic nerves were not included in the calculation.

Supplementary Table S3. The relationship between the surgical technique during radical trachelectomy and oncological and obstetrical outcomes.

		Attempt for pregnancy	P-value	Pregnancy rate	P-value	Live birth rate	P-value	Recurrence rate	P-value	Preterm delivery	P-value
**Abdominal radical trachelectomy**		513/1118 (45.89%)	< 0.001	207/513 (40.35%)	< 0.001	168/227 (74.00%)	0.592	51/1158 (4.4%)	0.554	64/146 (43.84%)	< 0.001
**Endoscopic radical trachelectomy**		182/388 (46.91%)		128/182 (70.33%)		134/172 (77.91%)		19/434 (4.38%)		36/134 (26.87%)	
**Vaginal radical trachelectomy**		420/755 (55,63%)		384/782 (49.1%)		351/468 (75.00%)		35/992 (3.53%)		119/315 (37.78%)	
**Uterine artery preservation**	yes	710/1375 (51.64%)	0.671	423/850 (49.76%)	0.455	403/516 (78.1%)	0.024	67/1651 (4.06%)	0.476	135/430 (31.4%)	0.089
	no	199/421 (47.3%)		175/370 (47.3%)		194/275 (70.55%)		32/663 (4.83%)		68/176 (38.64%)	
**Abdominal cerclage**	yes	1072/2167 (49.47%)	< 0.01	702/1464 (47.95%)	0.920	540/727 (74.23%)	0.940	97/2656 (3.65%)	0.313	234/717(32.64%)	0.786
	no	22/69 (31.9%)		10/22 (45.5%)		6/8 (75%)		4/56 (7.1%)		2/9 (22.2%)	
**Nerve sparing surgery**	yes	178/368(48.37%)	0.670	129/204 (63.24%)	0.041	82/103 (80.58%)	0.650	7/129 (5.43%)	0.718	31/98 (31.63%)	0.897
	no	159/342 (46.49%)		153/284 (53.87%)		191/249 (76.71%)		35/819 (4.27%)		71/230 (30.87%)	

The 'attempt for pregnancy rate' was defined as the rate of patients who declared trying for pregnancy. The 'pregnancy rate' was defined as the rate of patients who became pregnant. The 'live birth rate' was defined as the rate of live births after 24 weeks of pregnancy among all patients who have had RT. The 'pregnancy rate among attempt' that was defined as the pregnancy rate among patients who actually attempted to become pregnant. The 'preterm delivery rate' was calculated as the rate of deliveries before 37 weeks of gestation among all live births.

References

1. Kiss, S.L.; Fandi, A.; Cozlea, A.L.; Gheorghe, M.; Stanca, M.; Bacalbașa, N.; Moldovan, A.A.; Căpîlna, M.E. Abdominal Radical Trachelectomy as Fertility-Sparing Management for Early Stages of Cervical Cancer: Our Experience in 18 Cases. *Exp Ther Med* **2021**, *22*, 674, doi:10.3892/etm.2021.10106.

2. Wang, Y.; Peng, Y.; Lin, Z.; Yao, T. The Safety and Effectiveness of Preserving the Ascending Uterine Artery in a Modified Fertility-Sparing Abdominal Radical Trachelectomy. *Eur J Obstet Gynecol Reprod Biol* **2020**, *252*, 193–197, doi:10.1016/j.ejogrb.2020.06.053.

3. Mabuchi, S.; Kimura, T. Extraperitoneal Radical Trachelectomy With Pelvic Lymphadenectomy. *International Journal of Gynecological Cancer* **2017**, *27*, 537–542, doi:10.1097/IGC.0000000000000918.

4. Li, X.; Li, J.; Wen, H.; Ju, X.; Chen, X.; Xia, L.; Ke, G.; Tang, J.; Wu, X. The Survival Rate and Surgical Morbidity of Abdominal Radical Trachelectomy Versus Abdominal Radical Hysterectomy for Stage IB1 Cervical Cancer. *Ann Surg Oncol* **2016**, *23*, 2953–2958, doi:10.1245/s10434-016-5216-1.

5. van Gent, M.D.J.M.; van den Haak, L.W.; Gaarenstroom, K.N.; Peters, A.A.W.; van Poelgeest, M.I.E.; Trimbos, J.B.M.Z.; de Kroon, C.D. Nerve-Sparing Radical Abdominal Trachelectomy Versus Nerve-Sparing Radical Hysterectomy in Early-Stage (FIGO IA2-IB) Cervical Cancer: A Comparative Study on Feasibility and Outcome. *International Journal of Gynecologic Cancer* **2014**, *24*, 735–743, doi:10.1097/IGC.0000000000000114.

6. Lintner, B.; Saso, S.; Tarnai, L.; Novak, Z.; Palfalvi, L.; Del Priore, G.; Smith, J.R.; Ungar, L. Use of Abdominal Radical Trachelectomy to Treat Cervical Cancer Greater Than 2 Cm in Diameter. *International Journal of Gynecological Cancer* **2013**, *23*, 1065–1070, doi:10.1097/IGC.0b013e318295fb41.

7. Muraji, M.; Sudo, T.; Nakagawa, E.; Ueno, S.; Wakahashi, S.; Kanayama, S.; Yamada, T.; Yamaguchi, S.; Fujiwara, K.; Nishimura, R. Type II Versus Type III Fertility-Sparing Abdominal Radical Trachelectomy for Early-Stage Cervical Cancer: A Comparison of Feasibility of Surgical Outcomes. *International Journal of Gynecologic Cancer* **2012**, *22*, 479–483, doi:10.1097/IGC.0b013e31823fa7bd.

8. Wethington, S.L.; Cibula, D.; Duska, L.R.; Garrett, L.; Kim, C.H.; Chi, D.S.; Sonoda, Y.; Abu-Rustum, N.R. An International Series on Abdominal Radical Trachelectomy: 101 Patients and 28 Pregnancies. *Int J Gynecol Cancer* **2012**, *22*, 1251–1257, doi:10.1097/IGC.0b013e318263eee2.

9. Karateke, A.; Kabaca, C. Radical Abdominal Trachelectomy Is a Safe and Fertility Preserving Option for Women with Early Stage Cervical Cancer. *Eur J Gynaecol Oncol* **2012**, *33*, 200–203.

10. Li, J.; Li, Z.; Wang, H.; Zang, R.; Zhou, Y.; Ju, X.; Ke, G.; Wu, X. Radical Abdominal Trachelectomy for Cervical Malignancies: Surgical, Oncological and Fertility Outcomes in 62 Patients. *Gynecol Oncol* **2011**, *121*, 565–570, doi:10.1016/j.ygyno.2011.01.032.

11. Nishio, H.; Fujii, T.; Kameyama, K.; Susumu, N.; Nakamura, M.; Iwata, T.; Aoki, D. Abdominal Radical Trachelectomy as a Fertility-Sparing Procedure in Women with Early-Stage Cervical Cancer in a Series of 61 Women. *Gynecol Oncol* **2009**, *115*, 51–55, doi:10.1016/j.ygyno.2009.06.036.

12. Okugawa, K.; Yahata, H.; Sonoda, K.; Ohgami, T.; Yasunaga, M.; Kaneki, E.; Kato, K. Safety Evaluation of Abdominal Trachelectomy in Patients with Cervical Tumors ≥2 Cm: A Single-Institution, Retrospective Analysis. *J Gynecol Oncol* **2020**, *31*, doi:10.3802/jgo.2020.31.e41.

13. Kim, C.H.; Abu-Rustum, N.R.; Chi, D.S.; Gardner, G.J.; Leitao, M.M.; Carter, J.; Barakat, R.R.; Sonoda, Y. Reproductive Outcomes of Patients Undergoing Radical Trachelectomy for Early-Stage Cervical Cancer. *Gynecol Oncol* **2012**, *125*, 585–588, doi:10.1016/j.ygyno.2012.03.014.

14. Căpîlna, M.E.; Ioanid, N.; Scripcariu, V.; Gavrilescu, M.M.; Szabo, B. Abdominal Radical Trachelectomy: A Romanian Series. *International Journal of Gynecologic Cancer* **2014**, *24*, 615–619, doi:10.1097/IGC.0000000000000076.

15. Li, X.; Li, J.; Jiang, Z.; Xia, L.; Ju, X.; Chen, X.; Wu, X. Oncological Results and Recurrent Risk Factors Following Abdominal Radical Trachelectomy: An Updated Series of 333 Patients. *BJOG* **2019**, *126*, 1169–1174, doi:10.1111/1471-0528.15621.

16. Nakajima, T.; Kasuga, A.; Hara-Yamashita, A.; Ikeda, Y.; Asai-Sato, M.; Nakao, T.; Hayashi, C.; Takeya, C.; Adachi, K.; Tsuruga, T.; et al. Reconstructed Uterine Length Is Critical for the Prevention of Cervical Stenosis Following Abdominal Trachelectomy in Cervical Cancer Patients. *J Obstet Gynaecol Res* **2020**, *46*, 328–336, doi:10.1111/jog.14153.

17. Li, X.; Xia, L.; Li, J.; Chen, X.; Ju, X.; Wu, X. Reproductive and Obstetric Outcomes after Abdominal Radical Trachelectomy (ART) for Patients with Early-Stage Cervical Cancers in Fudan, China. *Gynecol Oncol* **2020**, *157*, 418–422, doi:10.1016/j.ygyno.2020.02.016.

18. Tamauchi, S.; Kajiyama, H.; Sakata, J.; Sekiya, R.; Suzuki, S.; Mizuno, M.; Utsumi, F.; Niimi, K.; Kotani, T.; Shibata, K.; et al. Oncologic and Obstetric Outcomes of Early Stage Cervical Cancer with Abdominal Radical Trachelectomy: Single-institution Experience. *Journal of Obstetrics and Gynaecology Research* **2016**, *42*, 1796–1801, doi:10.1111/jog.13100.

19. Nishio, H.; Fujii, T.; Sugiyama, J.; Kuji, N.; Tanaka, M.; Hamatani, T.; Miyakoshi, K.; Minegishi, K.; Tsuda, H.; Iwata, T.; et al. Reproductive and Obstetric Outcomes after Radical Abdominal Trachelectomy for Early-Stage Cervical Cancer in a Series of 31 Pregnancies. *Human Reproduction* **2013**, *28*, 1793–1798, doi:10.1093/humrep/det118.

20. Tokunaga, H.; Watanabe, Y.; Niikura, H.; Nagase, S.; Toyoshima, M.; Shiro, R.; Yokoyama, Y.; Mizunuma, H.; Ohta, T.; Nishiyama, H.; et al. Outcomes of Abdominal Radical Trachelectomy: Results of a Multicenter Prospective Cohort Study in a Tohoku Gynecologic Cancer Unit. *Int J Clin Oncol* **2015**, *20*, 776–780, doi:10.1007/s10147-014-0763-6.

21. Wang, Y.; Wang, A.; Zhan, J.; Guo, T. Curative Effect of Laparoscopic-Assisted Vaginal Radical Trachelectomy Combined with Pelvic Lymph Node Dissection on Early-Stage Cervical Cancer. *J BUON* **2021**, *26*, 684–690.

22. Johansen, G.; Lönnerfors, C.; Falconer, H.; Persson, J. Reproductive and Oncologic Outcome Following Robot-Assisted Laparoscopic Radical Trachelectomy for Early Stage Cervical Cancer. *Gynecol Oncol* **2016**, *141*, 160–165, doi:10.1016/j.ygyno.2016.01.028.

23. Ekdahl, L.; Paraghamian, S.; Eoh, K.J.; Thumuluru, K.M.; Butler-Manuel, S.A.; Kim, Y.T.; Boggess, J.F.; Persson, J.; Falconer, H. Long Term Oncologic and Reproductive Outcomes after Robot-Assisted Radical Trachelectomy for Early-Stage Cervical Cancer. An International Multicenter Study. *Gynecol Oncol* **2022**, *164*, 529–534, doi:10.1016/j.ygyno.2021.12.029.

24. Xu, M.; Huo, C.; Huang, C.; Liu, Y.; Ling, X.; Xu, G.; Lin, Z.; Lu, H. Round Ligament Suspension and Vaginal Purse-String Suture: Newly Optimized Techniques to Prevent Tumor Spillage in Laparoscopic Radical Trachelectomy for Cervical Cancer. *J Obstet Gynaecol Res* **2022**, *48*, 1867–1875, doi:10.1111/jog.15278.

25. Xu, M.; Huo, C.; Huang, C.; Wu, B.; Liu, Y.; Li, J.; Ling, X.; Xu, G.; Lin, Z.; Lu, H. Feasibility of the "Cuff-Sleeve" Suture Method for Functional Neocervix Reconstruction in Laparoscopic Radical Trachelectomy: A Retrospective Analysis. *J Minim Invasive Gynecol* **2022**, *29*, 673–682, doi:10.1016/j.jmig.2022.01.002.

26. Kanao, H.; Aoki, Y.; Fusegi, A.; Omi, M.; Nomura, H.; Tanigawa, T.; Okamoto, S.; Kurita, T.; Netsu, S.; Omatsu, K.; et al. Feasibility and Outcomes of "No-Look No-Touch" Laparoscopic Radical Trachelectomy for Early-Stage Cervical Cancer. *J Clin Med* **2021**, *10*, 4154, doi:10.3390/jcm10184154.

27. Saadi, J.; Minig, L.; Noll, F.; Saraniti, G.; Cárdenas-Rebollo, J.M.; Perrotta, M. Four Surgical Approaches to Cervical Excision During Laparoscopic Radical Trachelectomy for Early Cervical Cancer. *J Minim Invasive Gynecol* **2017**, *24*, 869–875, doi:10.1016/j.jmig.2017.04.010.

28. Saadi, J.M.; Perrotta, M.; Orti, R.; Salvo, G.; Giavedoni, M.E.; Gogorza, S.; Testa, R. Laparoscopic Radical Trachelectomy: Technique, Feasibility, and Outcomes. *JSLS* **2015**, *19*, e2013.00248, doi:10.4293/JSLS.2013.00248.

29. Kim, J.; Park, J.; Kim, D.; Kim, Y.; Kim, Y.; Nam, J. Fertility-sparing Laparoscopic Radical Trachelectomy for Young Women with Early Stage Cervical Cancer. *BJOG* **2010**, *117*, 340–347, doi:10.1111/j.1471-0528.2009.02446.x.

30. Chen, Y.; Xu, H.; Zhang, Q.; Li, Y.; Wang, D.; Liang, Z. A Fertility-Preserving Option in Early Cervical Carcinoma: Laparoscopy-Assisted Vaginal Radical Trachelectomy and Pelvic

Lymphadenectomy. *European Journal of Obstetrics & Gynecology and Reproductive Biology* **2008**, *136*, 90–93, doi:10.1016/j.ejogrb.2006.10.014.

31. Ekdahl, L.; Crusensvärd, M.; Reynisson, P.; Lönnerfors, C.; Persson, J. Quality of Life and Long-Term Clinical Outcome Following Robot-Assisted Radical Trachelectomy. *Eur J Obstet Gynecol Reprod Biol* **2021**, *267*, 234–240, doi:10.1016/j.ejogrb.2021.11.018.

32. Park, N.Y.; Chong, G.O.; Cho, Y.L.; Park, I.S.; Lee, Y.S. Total Laparoscopic Nerve-Sparing Radical Trachelectomy. *Journal of Laparoendoscopic & Advanced Surgical Techniques* **2009**, *19*, 53–58, doi:10.1089/lap.2007.0231.

33. Ebisawa, K.; Takano, M.; Fukuda, M.; Fujiwara, K.; Hada, T.; Ota, Y.; Kurotsuchi, S.; Kanao, H.; Andou, M. Obstetric Outcomes of Patients Undergoing Total Laparoscopic Radical Trachelectomy for Early Stage Cervical Cancer. *Gynecol Oncol* **2013**, *131*, 83–86, doi:10.1016/j.ygyno.2013.07.108.

34. Kucukmetin, A.; Biliatis, I.; Ratnavelu, N.; Patel, A.; Cameron, I.; Ralte, A.; Naik, R. Laparoscopic Radical Trachelectomy Is an Alternative to Laparotomy With Improved Perioperative Outcomes in Patients With Early-Stage Cervical Cancer. *International Journal of Gynecologic Cancer* **2014**, *24*, 135–140, doi:10.1097/IGC.0000000000000031.

35. Persson, J.; Imboden, S.; Reynisson, P.; Andersson, B.; Borgfeldt, C.; Bossmar, T. Reproducibility and Accuracy of Robot-Assisted Laparoscopic Fertility Sparing Radical Trachelectomy. *Gynecol Oncol* **2012**, *127*, 484–488, doi:10.1016/j.ygyno.2012.08.022.

36. Shinkai, S.; Ishioka, S.; Mariya, T.; Fujibe, Y.; Kim, M.; Someya, M.; Saito, T. Does Radical Trachelectomy (RT) during Pregnancy Have Higher Obstetrical and Oncological Risks than RT before Pregnancy? *Arch Gynecol Obstet* **2022**, *306*, 189–197, doi:10.1007/s00404-021-06327-w.

37. Plaikner, A.; Jacob, A.; Siegler, K.; Schneider, A.; Ragosch, V.; Barinoff, J.; Kohler, C. Modification of Dargent's Radical Vaginal Trachelectomy to Facilitate Ureteral Dissection: Description of Technique. *International Journal of Gynecological Cancer* **2020**, *30*, 1210–1214, doi:10.1136/ijgc-2020-001478.

38. Meglič, L.; Čavič, M.; Tomaževič, T.; Kobal, B.; Cvjetićanin, B.; Možina, A.; Barbič, M.; Smrkolj, Š. Laparoscopic Abdominal Cerclage after Radical Vaginal Trachelectomy. *Clin Exp Obstet Gynecol* **2017**, *44*, 343–346, doi:10.12891/ceog3192.2017.

39. Takada, S.; Ishioka, S.I.; Endo, T.; Baba, T.; Morishita, M.; Akashi, Y.; Mizuuchi, M.; Adachi, H.; Kim, M.; Saito, T. Difficulty in the Management of Pregnancy after Vaginal Radical Trachelectomy. *Int J Clin Oncol* **2013**, *18*, 1085–1090, doi:10.1007/s10147-012-0479-4.

40. Hertel, H.; Köhler, C.; Grund, D.; Hillemanns, P.; Possover, M.; Michels, W.; Schneider, A. Radical Vaginal Trachelectomy (RVT) Combined with Laparoscopic Pelvic Lymphadenectomy: Prospective Multicenter Study of 100 Patients with Early Cervical Cancer. *Gynecol Oncol* **2006**, *103*, 506–511, doi:10.1016/j.ygyno.2006.03.040.

41. Speiser, D.; Mangler, M.; Köhler, C.; Hasenbein, K.; Hertel, H.; Chiantera, V.; Gottschalk, E.; Lanowska, M. Fertility Outcome after Radical Vaginal Trachelectomy: A Prospective Study of

212 Patients. *International Journal of Gynecological Cancer* **2011**, *21*, 1635–1639, doi:10.1097/IGC.0b013e3182230294.

42. Burnett, A.F.; Roman, L.D.; O'Meara, A.T.; Morrow, C.P. Radical Vaginal Trachelectomy and Pelvic Lymphadenectomy for Preservation of Fertility in Early Cervical Carcinoma. *Gynecol Oncol* **2003**, *88*, 419–423, doi:10.1016/S0090-8258(02)00142-7.

43. Dargent, D.; Martin, X.; Sacchetoni, A.; Mathevet, P. Laparoscopic Vaginal Radical Trachelectomy: A Treatment to Preserve the Fertility of Cervical Carcinoma Patients. *Cancer* **2000**, *88*, 1877–1882, doi:10.1002/(SICI)1097-0142(20000415)88:8<1877::AID-CNCR17>3.0.CO;2-W.

44. Roy, M.; Plante, M. *Pregnancies after Radical Vaginal Trachelectomy for Early-Stage Cervical Cancer*; 1998;

45. Hauerberg, L.; Høgdall, C.; Loft, A.; Ottosen, C.; Bjoern, S.F.; Mosgaard, B.J.; Nedergaard, L.; Lajer, H. Vaginal Radical Trachelectomy for Early Stage Cervical Cancer. Results of the Danish National Single Center Strategy. *Gynecol Oncol* **2015**, *138*, 304–310, doi:10.1016/j.ygyno.2015.05.016.

46. van der Plas, R.C.J.; Bos, A.M.E.; Jürgenliemk-Schulz, I.M.; Gerestein, C.G.; Zweemer, R.P. Fertility-Sparing Surgery and Fertility Preservation in Cervical Cancer: The Desire for Parenthood, Reproductive and Obstetric Outcomes. *Gynecol Oncol* **2021**, *163*, 538–544, doi:10.1016/j.ygyno.2021.09.003.

47. Bernardini, M.; Barrett, J.; Seaward, G.; Covens, A. Pregnancy Outcomes in Patients after Radical Trachelectomy. *Am J Obstet Gynecol* **2003**, *189*, 1378–1382, doi:10.1067/S0002-9378(03)00776-2.

48. Wang, A.; Cui, G.; Jin, C.; Wang, Y.; Tian, X. Multicenter Research on Tumor and Pregnancy Outcomes in Patients with Early-Stage Cervical Cancer after Fertility-Sparing Surgery. *Journal of International Medical Research* **2019**, *47*, 2881–2889, doi:10.1177/0300060519845974.

49. Brătilă, E.; Brătilă, C.P.; Coroleuca, C.B. Radical Vaginal Trachelectomy with Laparoscopic Pelvic Lymphadenectomy for Fertility Preservation in Young Women with Early-Stage Cervical Cancer. *Indian Journal of Surgery* **2016**, *78*, 265–270, doi:10.1007/s12262-015-1351-3.

50. Rizzuto, I.; MacNab, W.; Nicholson, R.; Nalam, M.; Rufford, B. Less Radical Surgery for Women with Early Stage Cervical Cancer: Our Experience on Radical Vaginal Trachelectomy and Laparoscopic Pelvic Lymphadenectomy. *Gynecol Oncol Rep* **2019**, *28*, 65–67, doi:10.1016/j.gore.2019.03.005.

51. Covens, A.; Shaw, P.; Murphy, J.; DePetrillo, D.; Lickrish, G.; Laframboise, S.; Rosen, B. Is Radical Trachelectomy a Safe Alternative to Radical Hysterectomy for Patients with Stage IA-B Carcinoma of the Cervix? *Cancer* **1999**, *86*, 2273–2279, doi:10.1002/(SICI)1097-0142(19991201)86:11<2273::AID-CNCR15>3.0.CO;2-C.

52. Schlaerth, J.B.; Spirtos, N.M.; Schlaerth, A.C. Radical Trachelectomy and Pelvic Lymphadenectomy with Uterine Preservation in the Treatment of Cervical Cancer. *Am J Obstet Gynecol* **2003**, *188*, 29–34, doi:10.1067/mob.2003.124.

53. Kim, M.; Ishioka, S. ichi; Endo, T.; Baba, T.; Akashi, Y.; Morishita, M.; Adachi, H.; Saito, T. Importance of Uterine Cervical Cerclage to Maintain a Successful Pregnancy for Patients Who Undergo Vaginal Radical Trachelectomy. *Int J Clin Oncol* **2014**, *19*, 906–911, doi:10.1007/s10147-013-0631-9.

54. Alexander-Sefre, F.; Chee, N.; Spencer, C.; Menon, U.; Shepherd, J.H. Surgical Morbidity Associated with Radical Trachelectomy and Radical Hysterectomy. *Gynecol Oncol* **2006**, *101*, 450–454, doi:10.1016/j.ygyno.2005.11.007.

55. Plante, M.; Gregoire, J.; Renaud, M.C.; Roy, M. The Vaginal Radical Trachelectomy: An Update of a Series of 125 Cases and 106 Pregnancies. *Gynecol Oncol* **2011**, *121*, 290–297, doi:10.1016/j.ygyno.2010.12.345.

56. Shepherd, J.H.; Mould, T.; Oram, D.H. Radical Trachelectomy in Early Stage Carcinoma of the Cervix: Outcome as Judged by Recurrence and Fertility Rates. *BJOG* **2001**, *108*, 882–885, doi:10.1111/j.1471-0528.2001.00212.x.
